# Supplementary material for: Predicting permeation of compounds across the outer membrane of P. aeruginosa using molecular descriptors
Source: Commun Chem. 2024 Apr 12;7:84. doi: 10.1038/s42004-024-01161-y (PMC11015012; doi:10.1038/s42004-024-01161-y)
Supplement: Supplementary file 1 — Supplementary Information [file 42004_2024_1161_MOESM1_ESM.pdf]

Supplementary Information

Predicting the Permeability of Compounds across the Outer Membrane of *P. aeruginosa* Using Molecular Descriptors

P. D. Manrique[1,*], I. V. Leus[2], C. A. López[3], J. Mehla[2], G. Malloci[4], S. Gervasoni[4], A. V. Vargiu[4], R. Kinthada[5], L. Herndon[3], N. W. Hengartner[3], J. K. Walker[5], V. V. Rybenkov[2], P. Ruggerone[4], H. I. Zgurskaya[2], S. Gnanakaran[3,*]

[1] *Physics Department, George Washington University, Washington, DC 20052, USA*

[2] *Department of Chemistry and Biochemistry, University of Oklahoma, Norman, Oklahoma, USA*

[3] *Theoretical Biology and Biophysics Group, Los Alamos National Laboratory, Los Alamos, NM 87545, USA*

[4] *Department of Physics, University of Cagliari, Monserrato (CA), Italy*

[5] *Department of Pharmacology and Physiology, St Louis University, School of Medicine, St. Louis, Missouri, USA*

*Correspondence: pmanriq@gmail.com (PDM), gnana@lanl.gov (SG)

Contents

0 Supplementary Methods **5**
- 0.1 Experimental Methods and Chemical Syntheses . . . 5
  - 0.1.1 Principal component analysis of our library of compounds . . . 5
- 0.2 Computational Setup and Protocols for Computing Molecular Descriptors . . . 5
- 0.3 QSAR, QM, and MD calculations . . . 5
  - 0.3.1 *P. aeruginosa* OM set up for MD . . . 6
  - 0.3.2 Ensemble docking to MexB . . . 7
- 0.4 Hierarchical Clustering Implementation . . . 7
  - 0.4.1 Agglomerative clustering: generalities and applications . . . 7
  - 0.4.2 Physicochemical and permeation descriptors cluster around the type of observable . . 9
  - 0.4.3 Correlations in the number of contacts between compounds and key residues in MexB are primarily associated to residues proximity . . . 9

1 Supplementary Note 1 **11**
- 1.1 Full ranking of clusters according to predictability of OM permeation using the full set of descriptors . . . 11
  - 1.1.1 Our reduction algorithm and its robustness across combinations of descriptors 13
  - 1.1.2 Performance of alternative combination of descriptors from the top-9 clusters 16

2 Supplementary Note 2 **18**
- 2.1 High performance permeation prediction from different combinations of descriptors across testing samples . . . 18

3 Supplementary Note 3 **20**
- 3.1 Classification of active compounds according to predictability . . . 20
  - 3.1.1 Permeation class threshold based on empirical IC_{50} ratio . . . 20
  - 3.1.2 Parameter regions associated with the different permeation classes . . . 21
  - 3.1.3 Structural aspects of compounds in critical subgroups . . . 23

4 Supplementary Note 4 **24**
- 4.1 Descriptor ranges associated to weak and strong permeation . . . 24

List of Figures

S1 Contribution of each of the nine listed properties to the principal components representation up to third degree for the full set of 1260 compounds used in this study. The contribution is calculated from the eigenvectors of the covariance matrix. 5

S2 Finding the optimal number of clusters for each set of molecular descriptors and for the combined set following the L method 8

S3 Clustering characterization of physicochemical descriptors. A hierarchical clustering is computed with the ranked correlations of 73 physicochemical descriptors for each of the 1263 compounds derived from quantitative structure-activity relationship (QSAR) modeling, density functional quantum mechanical calculations (QM), and molecular dynamics (MD) simulations in water, as shown in the color bar at the right hand side. The calculation yields 24 clusters arranged by the dendrogram and illustrated by heatmap by means of the dissimilarity defined as $(1 - R_{i,j}^2)^{1/2}$, where R_{ij} is the ranked correlation between descriptors i and j. 10

S4 Hierarchical clustering characterization of the permeation descriptors revealing 5 dissimilar clusters grouped, for the most part, by physical quantity. 11

S5 Hierarchical clustering of docking descriptors showing a subset of those associated to contacts to residues in MexB. Our statistical analysis yields 21 clusters in total. For illustration purposes, we present 16 of them that group the number of contacts between the compound and specific residues in the DP_T (upper left) and AP_L (bottom left) pockets of MexB. There is a clear separation between the descriptors associated to residues in the DP_T and those in the AP_L, showing stronger similarities in the former, as shown the dendrogram (right panel). These residues depict clear correlations between proximity and cluster membership. 12

S6 Precision (left), recall (center), and F1 (right) testing results evaluated in the testing portion of the reduction algorithm presented in the main paper. Each orange circle points to the performance of a random combination of x compounds, while the horizontal green line is the evaluation metric when all descriptors are considered. Vertical blue line indicates the $x = 9$ mark. 15

S7 Detailed output of the model in the space of the 120 testing compounds. (a) Direct comparison between the model target class output and the data. The classification output is computed over several random splits of training/validation, hence the average class is reported and could be interpreted as the probability that a given compound is a strong permeator (class 1). (b) Equivalent target class out put but projected in the space of the top-2 descriptors (HB-MEM-INTER, and Δh-LIPID-A). Correct predictions (TP and TN) are shown combined in gray, while the false negatives are orange and the false positive in green. (c) Receiver operating characteristic (ROC) curve associated to our classification model (dark gray) compared to a fully random classification model (black dashed line). (d) Evaluation metrics of the classification model over the testing sample. 16

S8 Model performance across clusters. (a) Model's prediction accuracy using all combinations of descriptors from the top-3 clusters (clusters, 8, 3 and 7). (b) Prediction accuracy for all pairs of descriptors from the forth and fifth ranked clusters together with top-3 descriptors from the optimal combination. (c) Prediction accuracy for all pairs of the seventh and eighth ranked clusters alongside with the top-6 descriptors from the optimal set and descriptor ASAplus/ASA (ranked nineth among clusters). 17

S9 Frequency of identification of specific descriptors for the top-9 clusters across 51 random testing sets of 121 compounds. 18

S10 Accuracy score over 50 randomly assembled testing samples using the different combinations of descriptors as explained in the text: combination j, combination a, and combination b. Gray horizontal line indicates the 90% mark in accuracy. 20

S11 Active compounds classification according to predictability for different choices of IC_{50} ratio threshold. The size of the sets G, R and B, are shown in the top, middle, and bottom panel, respectively. A threshold of 0.5 maximizes the compounds in the set G while minimizing the sets R and B, as shown. 21

S12 Compounds of the set G projected over the nine descriptors highlighted by the reduction algorithm, as listed in the bottom left. In each of the scatter plots shown, red dots represent strong permeators, while blue are weak permeators. For each row (column) the y-(x-)axis represents a single descriptor, as shown. 22

S13 Projection of the 600 active compounds over the top-2 descriptors that predict permeation the best for the three predictive classification groups: set G (left panel), set R (central panel), and set B (right panel). 23

S14 Common structural motif (3-aminoquinoline) that is present in all compounds of this subgroup, which could help identify these compounds and prevent errors in their classification . . 24

S15 Complementary analysis of relevant descriptors for the remaining seven Tanimoto subgroups that contain relevant number of compound of sets R and B, as illustrated in Figure 5d of the main manuscript. In cases where the separation between weak and strong permeators is somewhat clear, it is marked by a class boundary black line found using an SVM algorithm. 27

S16 2-dimensional analysis of the parameter regions associated to weak (blue) and strong (red) permeation for four sets of highly accurate predictors found. Scatter plot at the left of each panel is equivalent to the density distribution plots at the right. Active compounds of the set G are shown on each panel. (a) Randicindex (QSAR), ASA_H (Å^2) (QSAR). (b) Δh-LIPID-A (permeation), HB-CORE-2 (permeation). (c) LUMO (QM), HB-TAILS (permeation). (d) Rotatable bonds (QSAR), ASA_H/ASA (QSAR). Straight line in the left panel of each figure results from the analysis done by the two-dimensional SVM algorithm. The specifics of the function is given in Table S7. 28

S17 3-dimensional analysis of the parameter regions associated to weak (blue) and strong (red) permeation for four sets of highly accurate predictors found. All 605 active compounds are shown on each panel. (a) PHE615_B (docking), Δh-CORE-2 (permeation), ASA_H/ASA (QSAR). (b) Δs-IL-GLY (permeation), Total charge (QSAR), ASA_H(Å^2)(QSAR). (c) PHE615_B (docking), Δh-LIPID-A (permeation), ASAplus/ASA (QSAR). (d) HOMO (QM), HB-MEM-INTER (permeation), ASA_H/ASA (QSAR). Accuracy score a_0 for each panel is also shown. Gray plane results from the analysis done by the three-dimensional SVM algorithm. The specifics of the function is given in Table S8. 29

List of Tables

S1 Ranking number (R#) of individual clusters according to permeation predictability, their cluster number (C#), and the descriptors that belong to each of cluster. 14

S2 Ranking of clusters c_j up to top-21, where we compare the ordering when using a fixed testing sample, with a ranking produced when the testing sample changes randomly on every single iteration. The ordering of the clusters is robust to these changes for the most part. 14

S3 Example of the variety of combinations of descriptors from the top-9 clusters identified that lead to maximal accuracy for different random testing samples. Since three clusters in the top-9 contain only one (c_8 and c_6) or two descriptors (c_{36}), the table lists those for clusters of size greater than 2 only. . 19

S4 Comparison of the different evaluation metrics for different combination of predictability sets: all sets, G+R, G+B, and R+B. For each combination set we show the average metric across 100 randomly assembled testing samples. 21

S5 Individual descriptor ranges associated with strong (SP) and weak (WP) OM permeation for the top-60 descriptors according to the accuracy score of all active compounds. For each ranking descriptor r belonging to cluster c_j, the threshold t_c separating the target classes, is listed in the measurement units of the descriptor. Column L|R indicates whether WP are associated with descriptor values smaller than t_c and hence SP associated with values larger than t_c (entry W|S), or vice versa (entry S|W). The evaluation metrics of positive predictive value (PPV), negative predictive value (NPV) and accuracy (a_0) are listed for each descriptor for the compounds of the set G only and for all active compounds, as indicated. 25

S6 Individual descriptor ranges associated with strong (SP) and weak (WP) OM permeation for the descriptors below the top-60 according to the accuracy score of all active compounds. For each ranking descriptor r belonging to cluster c_j, the threshold t_c separating the target classes, is listed in the measurement units of the descriptor. Column L|R indicates whether WP are associated with descriptor values smaller than t_c and hence SP associated with values larger than t_c (entry W|S), or vice versa (entry S|W). The evaluation metrics of positive predictive value (PPV), negative predictive value (NPV) and accuracy (a_0) are listed for each descriptor for the compounds of the set G only and for all active compounds, as indicated. 26

S7 Top-40 pairs of descriptors and associated ranges with strong (SP) and weak (WP) OM permeation according to the accuracy score of all active compounds. The trained classification model over the compounds in the set G follow the linear equation $y = ax + b$, where y and x are the axes of descriptor i and j, respectively, and they are listed in the measurement units of the descriptors. Column L|R indicates whether WP are associated with descriptor values to the left of the trained linear model and hence SP associated with values to the right of the linear model (entry W|S), or vice versa (entry S|W). The evaluation metrics of positive predictive value (PPV), negative predictive value (NPV) and accuracy (a_0) are listed for each descriptor for the compounds of the set G only and for all active compounds, as indicated. 30

S8 Top-40 groups of three descriptors and associated ranges with strong (SP) and weak (WP) OM permeation according to the accuracy score of all active compounds. The trained classification model over the compounds in the set G follow the equation $ax + by + cz + d = 0$, where x, y, and z are the axes of descriptors i, j, and k, respectively, and they are listed in the measurement units of the descriptors. The evaluation metrics of positive predictive value (PPV), negative predictive value (NPV) and accuracy (a_0) are listed for each descriptor for the compounds of the set G only and for all active compounds, as indicated. 31

0 Supplementary Methods

0.1 Experimental Methods and Chemical Syntheses

The experimental set-up has been reported before [1, 2]. Briefly, *P. aeruginosa* cells were grown in Luria Bertani Broth (LB) (10 g tryptone, 5 g yeast extract, 5 g NaCl per liter, pH 7.0) at 37° C with shaking. Inhibitory concentration (IC_{50}) determination was carried out using the 2-fold broth dilution method. Two independent experiments were carried out. The expression of the Pore was induced at $OD_{600} = 0.3 - 0.4$ by addition of 0.1 mM IPTG. Chemical structures of the assembled library of 1260 compounds and the measured IC_{50} values are available upon request.

0.1.1 Principal component analysis of our library of compounds

As mentioned in the main paper, the library is comprised of 16 different structural groups coming from different sources, including known antibiotics. A principal components decomposition using nine basic properties properties is shown in Fig. 1d of the main paper. Figure S1, shows the contribution of each of these nine properties to the first three principal components. This is calculated from the eigenvectors of the covariance matrix of the standardized data (i.e., after a z-score normalization). The absolute value of the entry of each eigenvector is scaled so that the sum is 100, i.e., a percentage. As shown, the first principal component has a rather similar contribution of each of the properties. On the other hand, the corresponding eigenvalues provide information about the variance held by each degree of the principal components. In Fig. 1d of the main paper, we show the percentage held by the first three.

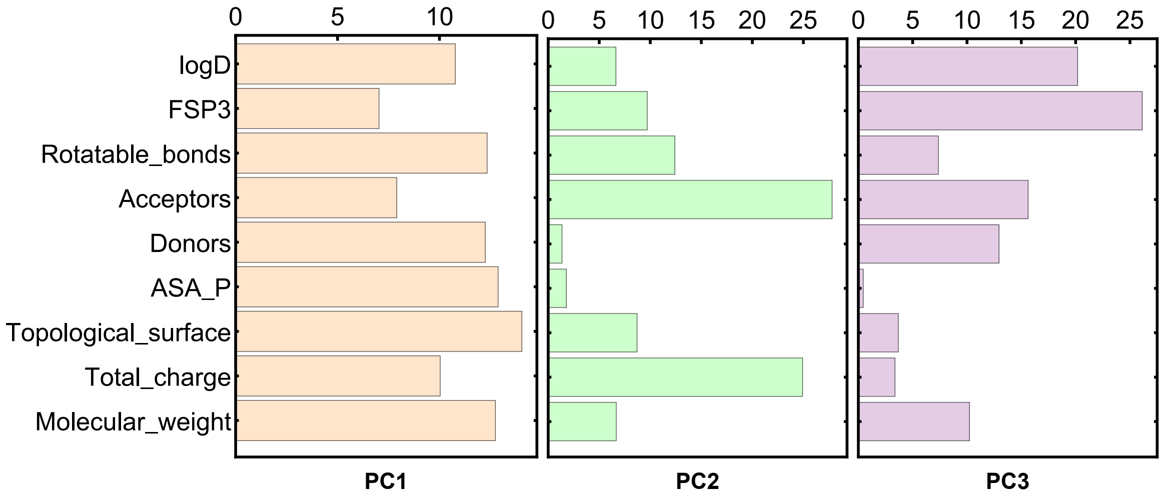

Figure S1: Contribution of each of the nine listed properties to the principal components representation up to third degree for the full set of 1260 compounds used in this study. The contribution is calculated from the eigenvectors of the covariance matrix.

0.2 Computational Setup and Protocols for Computing Molecular Descriptors

0.3 QSAR, QM, and MD calculations

For each compound we considered the protonation/charge state most populated at physiological pH. We used the ChemAxon's Marvin suite of programs [3] to obtain standard 1-2-3D descriptors used in QSAR studies (e.g., numbers of heavy atoms, rotatable bonds, H-bond donors/acceptors, van der Waals volume and surface, etc. see [1]). The geometry of the major microspecies has been used to perform QM calculations with the Gaussian16 package [4] as previously described [5]. Employing a polarizable continuum model to mimic the effect of water solvent we optimized the ground-state structure and performed full vibrational analysis, obtaining real frequencies in all cases. On the optimized geometry, we performed single-point energy

calculations in vacuum to generate the atomic partial charges fitting the molecular electrostatic potential. Under the constraint of reproducing the electric dipole moment of the molecule, we used the Merz-Kollman scheme [6]. Atomic partial charges were generated through the two-step restrained electrostatic potential method [7] implemented in the AnteChamber package [8]. With this program we derived general Amber force field (GAFF) parameters [9]. QM descriptors associated with the ground-state optimized structure include static polarizabilities, frontier molecular orbital energies, permanent dipole moment, and rotational constants. For each compound, we performed 1-μs-long all-atom MD simulation in explicit water solution (0.1 M KCl) using the Amber18 package as described before [5]. From MD simulations, we obtained structural and dynamic features of the compounds investigated by means of the CPPTRAJ program [10]. The number and population of structural clusters were determined using a hierarchical agglomerative algorithm [11].

0.3.1 *P. aeruginosa* OM set up for MD

The initial coordinates of the Outer membrane (OM) corresponding to the *P. aeruginosa* Gram-negative bacteria were downloaded from http://dqfnet.ufpe.br/biomat/software.html. The model has been parameterized in line with the GLYCAM force field[18] and parameters are adapted to run in the GROMACS[19] molecular dynamics engine. Briefly, the OM consists of an inner leaflet composed of 1,2-dipalmitoyl-sn-glycero-3-phosphoethanolamine (DPPE) and an outer leaflet composed of a truncated LPS structure. The membrane is fully solvated using the TIP3P water[20] model and anionic charges in the LPS molecules are counter balanced with CA++ cations. A schematic representation of the model is provided in Fig. 1e in the main paper and more details about its parameterization can be found in the original work[21].

Compounds were represented using the Amber force field. First, we optimized the ground-state structure of each compound employing a polarizable continuum model [22] as to mimic the effect of water solvent particularly to avoid formation of strong intramolecular H-bonds. This geometry was confirmed performing a full vibrational analyses, obtaining real frequencies in all cases. On the optimized geometry, we then performed single-point energy calculations in vacuum to generate the atomic partial charges fitting the molecular electrostatic potential. Under the constraint of reproducing the electric dipole moment of the molecule, we used the Merz-Kollman scheme [23] to construct a grid of points around the molecule. Atomic partial charges were then generated through the two-step restrained electrostatic potential method [24] implemented in the AnteChamber package [25]. Using this program, we derived general Amber force field (GAFF) parameter[26], which were transformed into GROMACS input files using the antechamber python parser interface (ACPYPE) tool[27].

MD protocol for computing the permeation descriptors In order to screen the molecular descriptors corresponding to the permeation along the OM membrane, each drug was placed into seven different molecular environments corresponding to specific regions along the normal of the OM (Fig. 1e in the main paper). These regions were explicitly selected in order to cover the influence of both the inner (DPPE) and outer leaflet (LPS) of the OM. Thus, seven independent simulations per drug were necessary in order to recapitulate the influence of the OM into the permeation process. The whole procedure was automated via a series of bash scripts, which iteratively connected the pulling code and energy minimization in GROMACS[19].

All simulations were run with the GROMACS 5.4.1 molecular dynamics engine[19] with a time step of 2 fs. The LINCS algorithm[28] was applied to constrain all bond lengths with a relative geometric tolerance of 10^{-4}. In line with its original parameterization, short-range interactions (vdW and Coulomb) were calculated using a cut-off scheme of 0.9 nm, which were evaluated based on a pair-list recalculated every five time steps. Long-range interactions were handled using a reaction field[29] correction with a permittivity dielectric constant of 66. After initial set-up, each system was energy minimized using 3000 steps of conjugated gradient, followed by a thermal equilibration of 1 ns. A harmonic potential of 1000

kJ mol-2, along the Z vector connecting the center of mass (COM) of the drug and the OM of the membrane was applied in order to maintain the relative position of the drug with respect to each of the seven defined regions of the membrane (Fig. 1). During equilibration, bilayers were coupled to 1.0 bar using a Berendsen barostat[30] through a semi-isotropic approach with relaxation time of 1.0 ps. Afterwards, production runs were coupled using a Parrinello barostat[31] algorithm and a constant temperature of 310K was maintained by weak coupling of the solvent and solute separately to a velocity-rescaling[32] scheme with a relaxation time of 1.0 ps. Production simulations were run for 20 ns and trajectories were saved each 20 ps.

A total of 8841 (176 μs) trajectories were analyzed using in-home developed bash scripts, which were directly interconnected to the in-built GROMACS tools. Thus, for each simulation the following molecular descriptors were evaluated (Fig. 1(e) in the main paper): Number of hydrogen bonds between the drug with its first solvation shell (HB-WATER), number of hydrogen bonds between the drug and the surrounding OM environment (HB), lateral mean squared displacement of the Drug (Δxy), Total enthalpic component of interaction between drug and surrounding environment (Δh),and total cummulative entropy of the drug (Δs). All these analysis were carried with the in-built analysis tool set provided in GROMACS.

0.3.2 Ensemble docking to MexB

Molecular docking calculations were performed using the AutoDock Vina package [12]. The program was used with default settings except for the exhaustiveness parameter which was set to 1024 (default of 8). Protein and ligand input files were prepared with AutoDock Tools [13]. Flexibility of docking partners was considered indirectly by using the ensemble of conformations. In particular, for each compound we used 10 different cluster representatives extracted from MD simulations in explicit water solution, while for MexB, we considered 6 conformations, including available X-ray crystal structures (PDB Ids 2V50, 3W9I, and 3W9J) [14, 15] and MD snapshots extracted from MD simulations [16]. For each docking run, we retained the top 10 docking poses. Following Ref. [17] we performed two sets of guided docking runs into the two major binding pockets of MexB: the access pocket of the access monomer (AP) and the deep binding pocket of the binding monomer (DP). In each case, the docking search was performed within a cubic volume of $40\times40\times40$ Å^3 centered in the center of mass of the pocket. The interaction between compounds and MexB was quantified by means of a statistical analysis of all poses, yielding about 60 descriptors. These descriptors include average binding affinities (according to the docking scoring function) as well as the total number of contacts with single residues lining the two pockets (see Table S1).

0.4 Hierarchical Clustering Implementation

0.4.1 Agglomerative clustering: generalities and applications

This is an unsupervised statistical technique that uses correlations among random variables to form groups (or clusters) of highly correlated quantities, resulting in clusters that are highly dissimilar from one another. This is a bottom-up technique that starts with clusters formed by a single random variable. Then the correlations coefficients among all the pairs are computed and ranked. The pair with the lowest dissimilarity measure is merged together into a cluster of size two. The dissimilarity D_{ij} is defined as the square-root of one minus the square of the correlation coefficient between the pair i and j:

$$D_{ij} = \sqrt{1 - R_{ij}^2}, \tag{1}$$

where, R_{ij} is the correlation coefficient between variables i and j. Subsequently, all correlation coefficients are computed again treating the cluster of two as a single variable in which the resultant correlation between the pair and another variable is derived as the average of the correlation with each member of the cluster individually. Then, the dissimilarity measures among all groups are ranked and the pair with the lowest one

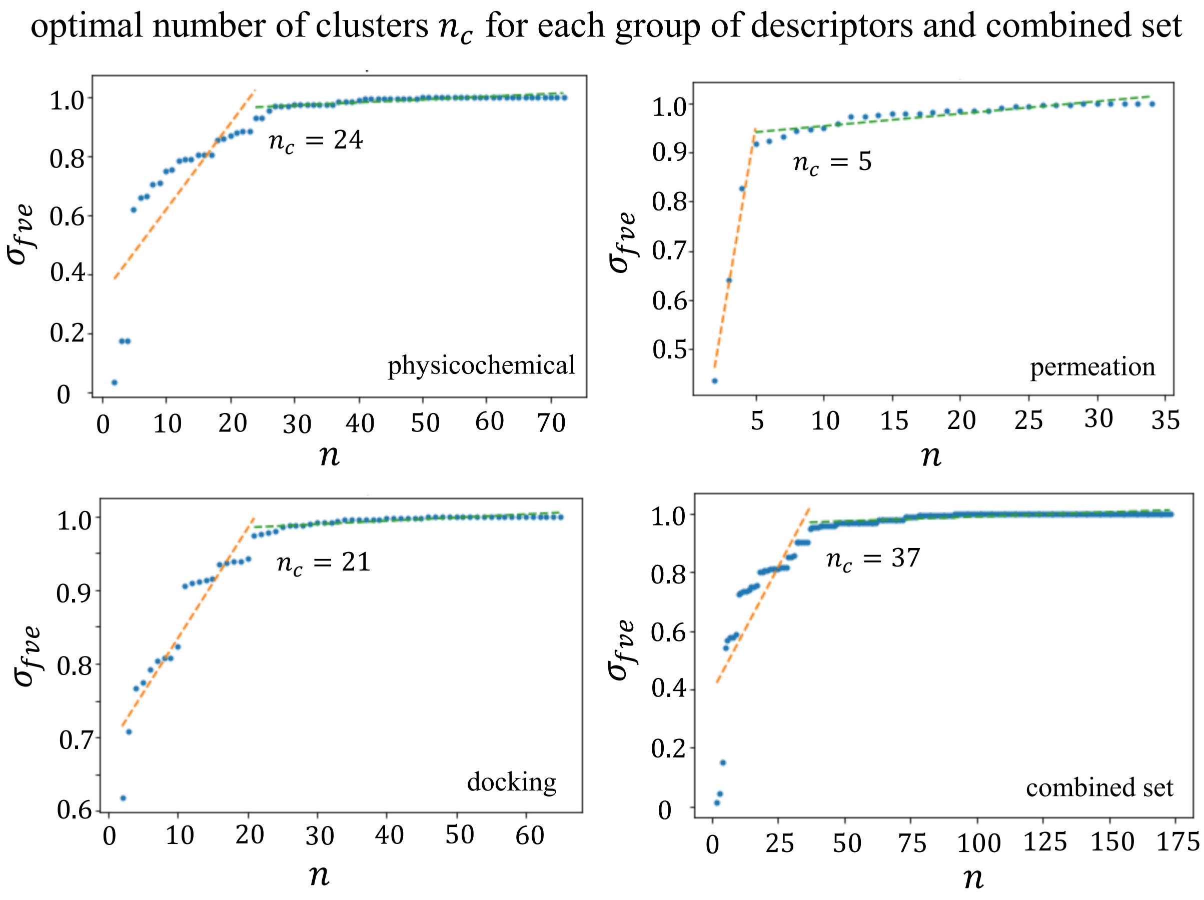

Figure S2: Finding the optimal number of clusters for each set of molecular descriptors and for the combined set following the L method

is merged into a larger cluster. This process is repeated until only one cluster remains.

In our analysis we implement the ranked correlations coefficients consisting on replacing the value of the random variable (i.e., molecular descriptor of the compound) for the low-to-high rank of such value within the distribution. For example, for the molecular property of molecular weight, the lightest compound would have a rank of one, the second lightest a rank of two, and so one. We do the same procedure for all descriptors. Then, the ranked correlations are calculated by computing the Pearson correlation coefficient over the list of ranked values.

In order to determine the optimal number of clusters we use the fractional variance explained defined as the ration of the variance between groups (i.e., points residing in different clusters) to the total variance (i.e., all points):

$$\sigma_{fve} = \frac{\sigma_{between\_groups}}{\sigma_{total}} = \frac{\sum_{ij|c_i \neq c_j} D_{ij}^2}{\sum_{ij} D_{ij}^2} \tag{2}$$

This quantity increases as the number of clusters decreases and then stabilizes, which points to an appropriate number of clusters. At this point variance from within clusters is small enough hinting at a relative closeness among points within clusters and otherwise for points in different clusters. The L method [] is employed to identify the optimal number of clusters n_c. First we create the list of the fractional variance explained σ_{fve} vs the number of clusters n. For each candidate number n we find the best straight line fit of all points before and after n, and compute the weighted sum of the root mean square error (RMSE) associated to the fits. The value of n that minimizes RMSE corresponds to the point in which the variance

stopped increasing as a function of the number of clusters. We consider this point as the optimal number of clusters. Figure S2 illustrates the application of this technique to determine the optimal number of clusters for each set of descriptors independently as well as for the combined set.

0.4.2 Physicochemical and permeation descriptors cluster around the type of observable

We compute non-linear hierarchical clustering to the group of 73 physicochemical descriptors, as well as, to the group of 35 permeation descriptors using the ranked correlations and the full set of compounds (see Fig 1). For both cases we find that the resultant clusters are formed around descriptors that quantify similar observables. This is not surprising for the physicochemical descriptors given that these quantities measure intrinsic properties of the compound (e.g., total charge), or they were computed in simple water environments. Fig S3 illustrates the classification of the 73 physicochemical descriptors in 24 clusters. The largest of them comprises 32 descriptors, which quantify properties akin to the size of the compound (e.g., molecular weight, atom count, and volume), together with positive, negative, and water accessible surface areas. These are mostly QSAR and QM descriptors. Slightly less correlated, but still within the same large cluster we find descriptors computed via MD simulations in water environments. These include the average value of the minimal projection area associated to the configurations explored by the molecule in the MD trajectory, and the average value of the root mean square fluctuation (RMSF) of the atomic positions. On the other hand, the remaining clusters are comprised by between one and five descriptors. These clusters group additional compounds properties such as charge and molecular orbitals, partition coefficients, shape, surface, and aromatic properties (full list given in the SM). Most of these smaller clusters contain only one type of descriptor (either MD, or QSAR or QM), and highly dissimilar among each other as depicted in the heatmap and dendrogram of Fig S3.

On the other hand, the permeation descriptors were computed through extensive all-atom MD simulations in seven different regions of the simulated OM of P. aeruginosa. These regions are characterized by having contrasting chemical properties and dissimilar compounds interact very differently with each of the region. In spite of this contrast, our clustering analysis groups together descriptors quantifying the same type of observable (e.g., entropy) measured at the different OM regions. This is in contrast with our analysis of the docking descriptors where it was found that the region where these properties were computed defined the cluster these belong to. Permeation descriptors group according to observable rather than the region. The only exception found is the number of hydrogen bonds between the compound and surrounding water molecules in the hydrophobic tails region, as well as, the lipid A sub-region. Our analysis finds that these descriptors are statistically more akin to the interaction energy (i.e., enthalpy Δh) between the compound and the OM than to the hydrogen bonds in the other regions. Moreover, hierarchical relationships among neighboring regions of the OM are found when we look at highly correlated descriptors belonging to the same cluster. The clearest example is the clustering of the entropy values, where we find a hierarchy of correlations among descriptors that resemble almost perfectly the neighboring regions in the OM. The most correlated pair is the entropies associated with the two outer sub-regions (SOL and HEAD), which in turn, is highly correlated to the third outer region of Glycerol. The resultant group is statistically more akin to the hydrophobic tails, forming a group that is more alike to lipid A. Finally, the resultant group is associated to the core regions. The high correlation between an observable measured in neighboring regions of the OM can be found in all clusters.

0.4.3 Correlations in the number of contacts between compounds and key residues in MexB are primarily associated to residues proximity

A statistical analysis of the docking descriptors using hierarchical clustering and rank correlations results in 21 clusters of different sizes ranging from single descriptor clusters and up to one cluster comprised of 13 descriptors. This is represented in the dendrogram of Fig S5. As reasonably found, descriptors associated

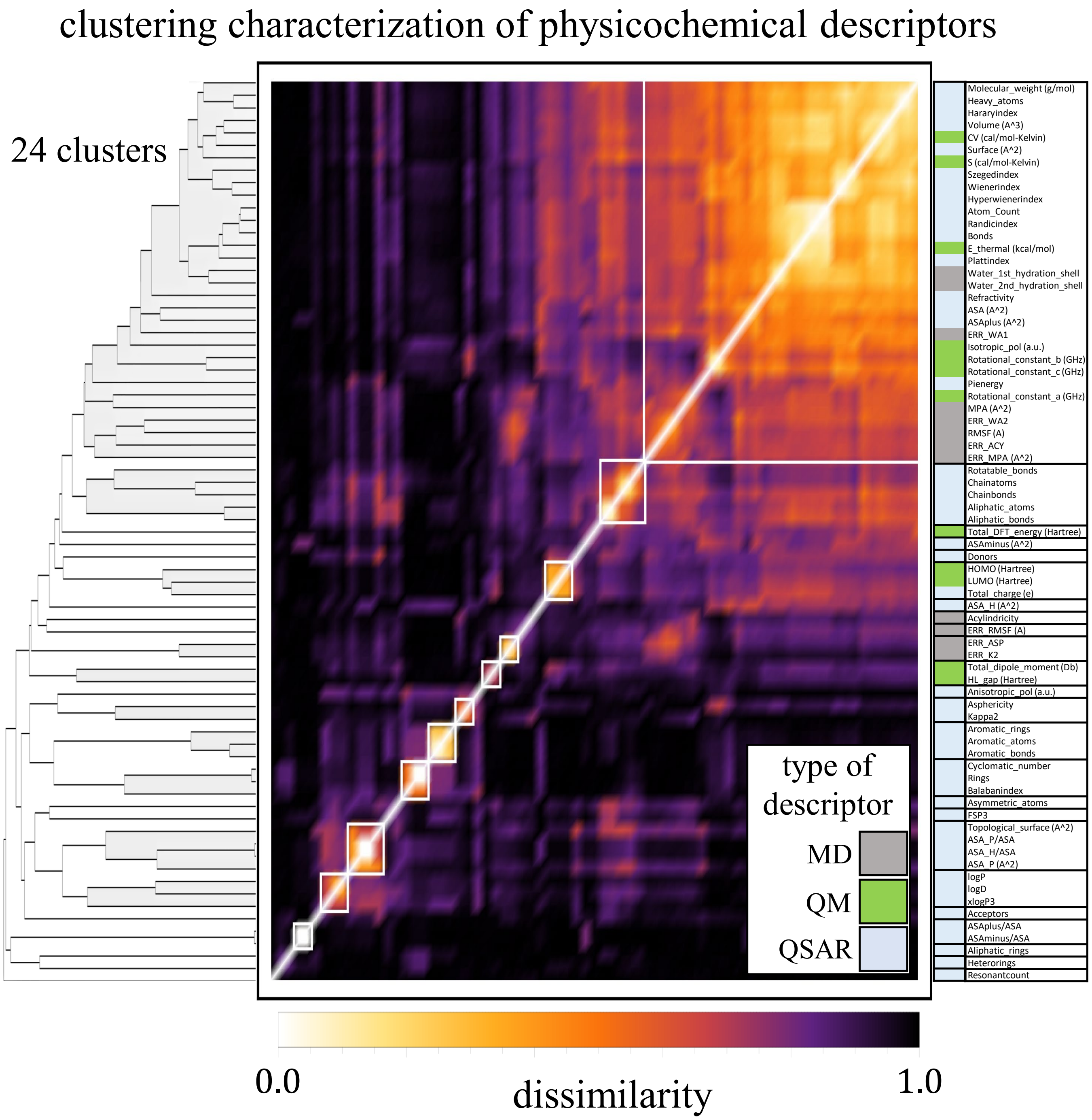

Figure S3: Clustering characterization of physicochemical descriptors. A hierarchical clustering is computed with the ranked correlations of 73 physicochemical descriptors for each of the 1263 compounds derived from quantitative structure-activity relationship (QSAR) modeling, density functional quantum mechanical calculations (QM), and molecular dynamics (MD) simulations in water, as shown in the color bar at the right hand side. The calculation yields 24 clusters arranged by the dendrogram and illustrated by heatmap by means of the dissimilarity defined as $(1 - R_{i,j}^2)^{1/2}$, where R_{ij} is the ranked correlation between descriptors i and j.

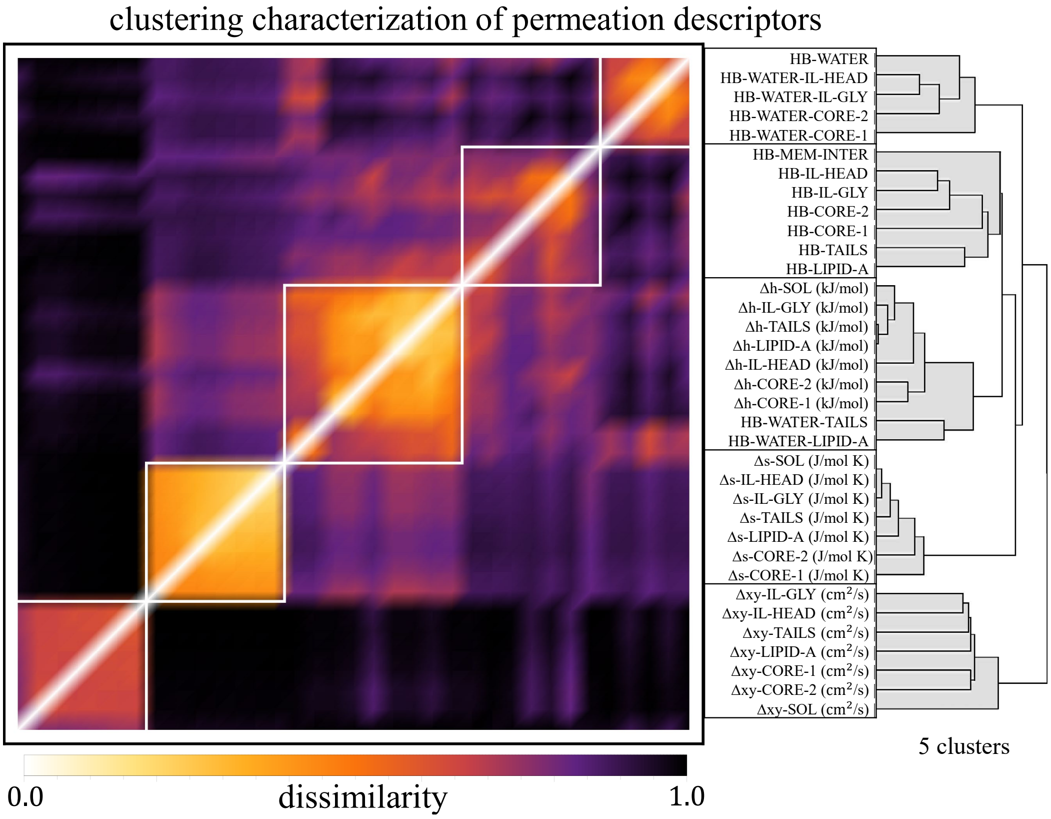

Figure S4: Hierarchical clustering characterization of the permeation descriptors revealing 5 dissimilar clusters grouped, for the most part, by physical quantity.

to DPB tend to cluster together and are highly dissimilar statistically from those associated to APA, which also clustered together among themselves. These two highly dissimilar groups break further into clusters of descriptors, some of which quantify the number of contacts to specific residues, while other the access and binding energies. A direct examination of the clusters containing the descriptors that quantify the contacts to key residues and the structure of the APA and DPB protomers, clustered residues tend to be in close proximity to each other within the protomer. The residues contained in the clusters are not characterized by similar physicochemical properties, but interestingly, the clusters also identify different sub regions of both of the pockets. For example, those in the DPB are close to the hydrophobic trap and appear to line a region in which compounds can fit well. In fact, the group of residues in the DPB are all hydrophobic, which is also consistent with what is expected since hydrophobicity is a common feature of good substrates/inhibitors of the transporter [36, 37]. On the other hand, those of the APA represent the sub-region just before entrance to the DPB, close to the G-loop separating the two pockets. In a like manner, we also find strong correlations among descriptors quantifying the average binding affinity (as predicted by docking) in the access and distal pockets however they are within the same cluster.

1 Supplementary Note 1

1.1 Full ranking of clusters according to predictability of OM permeation using the full set of descriptors

Table S1 lists all of the clusters, their low-to-high ranking according to prediction of permeability, and the descriptors that comprise each of the clusters as illustrated in the Figure 3 of the main paper. Complementary of this ranking, we repeat the process but we now randomly produce a new training and testing sample after each model iteration. The ranking up to top-21 is shown in Table S2. As shown, the top-9 clusters

hierarchical clustering of **docking** descriptors

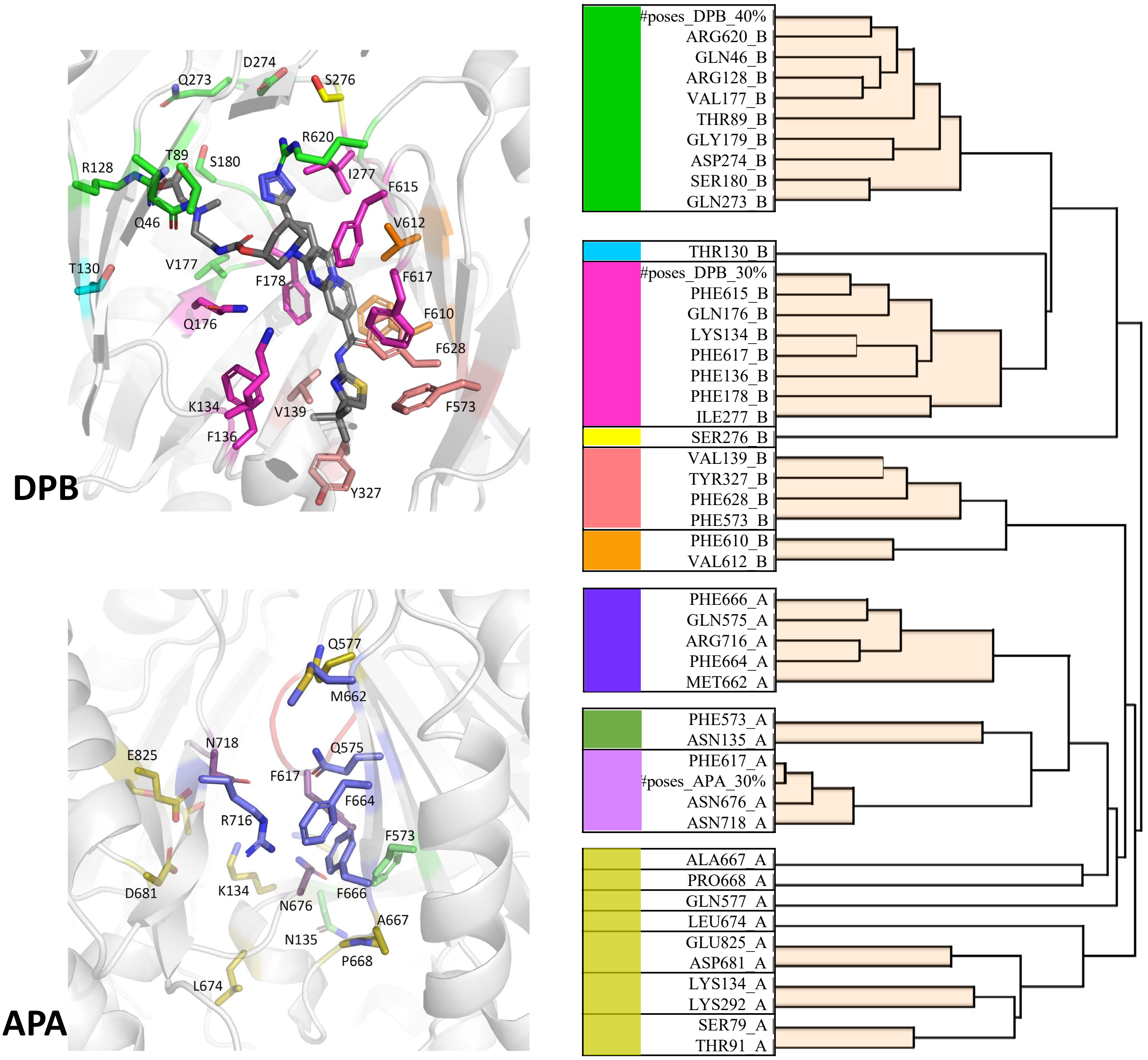

Figure S5: Hierarchical clustering of docking descriptors showing a subset of those associated to contacts to residues in MexB. Our statistical analysis yields 21 clusters in total. For illustration purposes, we present 16 of them that group the number of contacts between the compound and specific residues in the DP_T (upper left) and AP_L (bottom left) pockets of MexB. There is a clear separation between the descriptors associated to residues in the DP_T and those in the AP_L, showing stronger similarities in the former, as shown the dendrogram (right panel). These residues depict clear correlations between proximity and cluster membership.

remains stable and their relevance is not dependent in the specifics of the testing sample employed for the data analysis.

1.1.1 Our reduction algorithm and its robustness across combinations of descriptors

Evaluation metrics: definitions The model evaluation metrics computed in this work are based on combinations of the output from the traditional confusion matrix, which compares the fruitfulness of the prediction (i.e., true or false), with the binary classification (class 1 or class 0) of the real data. Hence, each prediction outcome can be classified as either true positive (TP, or class 1 correctly identified), true negative (TN, or class 0 correctly identified), false positive (FP, or a real class 0 identified as class 1), or false negative (FN, or real class 1 identified as class 0). The accuracy (a_0) is the ratio of correct predictions to all predictions, i.e., the fraction of correct predictions:

$$a_0 = \frac{TP + TN}{TP + TN + FP + FN}. \tag{3}$$

Similarly, the classification error (e_0) is defined as the opposite to the accuracy, which is the ratio of the incorrect predictions to all predictions, i.e., the fraction of incorrect predictions:

$$e_0 = 1 - a_0 = \frac{FP + FN}{TP + TN + FP + FN}. \tag{4}$$

In addition, metrics targeted to reduce specific error outcomes provide different angles of a given classification model. For example, recall (r_0) calculates the fraction of all positive instances that are correctly identified, and hence it is also known as the true positive rate (TPR). Its purpose is to minimize the false negative outputs:

$$r_0 = \frac{TP}{TP + FN} \tag{5}$$

The opposite of recall, is the specificity (s_0), which quantifies the fraction of all negative instances that the classifier identify as positive. Hence, it is known also as the false positive rate (FPR):

$$s_0 = \frac{FP}{TN + FP} \tag{6}$$

Moreover, the measure of precision is targeted to minimize false positives, and it is also known as the positive predictive value (PPV). It quantifies the fraction of positive predictions that are real:

$$PPV = \frac{TP}{TP + FP} \tag{7}$$

An equivalent measure to precision, but targeted to quantify the fraction of negative predictions that are real, is known as the negative predictive value (NPV):

$$NPV = \frac{TN}{TN + FN}. \tag{8}$$

Often, there is a tradeoff between precision and recall. Hence, a quantify known as $F1$ that effectively combines these two measurements as the harmonic mean of them is defined as:

$$F1 = \frac{2TP}{2TP + FN + FP}. \tag{9}$$

R#	C#	Descriptors
37	29	QSAR: Aliphatic_rings
36	27	D: LEU674_A
35	17	QSAR: Acceptors
34	22	D: ALA667_A
33	31	QSAR: Aromatic_atoms, QSAR: Aromatic_bonds, QSAR: Aromatic_rings
32	23	D: PRO668_A
31	33	P: Δxy-IL-HEAD (cm^2/s), P: Δxy-IL-GLY (cm^2/s), P: Δxy-TAILS (cm^2/s), P: Δxy-LIPID-A (cm^2/s), P: Δxy-CORE-1 (cm^2/s), P: Δxy-CORE-2 (cm^2/s)
30	37	QSAR: Resonantcount
29	28	QSAR: Heterorings
28	26	D: SER79_A, D: THR91_A, D: LYS134_A, D: LYS292_A, D: ASP681_A, D: GLU825_A
27	14	D: SER276_B
26	20	D: #poses_APA_40%, D: GLN575_A, D: MET662_A, D: PHE664_A, D: PHE666_A, D: ARG716_A
25	21	MD: ERR_ASP, MD: ERR_K2
24	35	D: GLN577_A
23	25	MD: #poses_DPB_20%
22	30	QSAR: Rings, QSAR: Balabanindex, QSAR: Cyclomatic_number
21	34	P: Δxy-SOL (cm^2/s)
20	21	MD: Asphericity, MD: Kappa2
19	11	QM: HL_gap (Hartree)
18	19	D: #poses_APA_20%, D: #poses_APA_30%, D: ASN135_A, D: PHE573_A, D: PHE617_A, D: ASN676_A, D: ASN718_A
17	15	QSAR: Asymmetric_atoms
16	5	QM: Total_DFT_energy (Hartree)
15	24	D: VAL139_B, D: TYR327_B, D: PHE573_B, D: PHE610_B, D: VAL612_B, D: PHE628_B
14	10	MD: Acylindricity
13	16	QSAR: FSP3
12	32	QSAR: Anisotropic_pol (a.u.)
11	14	QSAR: xlogP3, QSAR: logP, QSAR: logD
10	9	MD: ERR_RMSF (Å)
9	36	QSAR: ASAplus/ASA, QSAR: ASAminus/ASA
8	1	QSAR: Molecular_weight (g/mol), QSAR: Atom_Count, QSAR: Heavy_atoms, QM: Rotational_constant_b (GHz), QM: Rotational_constant_c (GHz), QSAR: Volume (Å^3), MD: Water_1st_hydration_shell, MD: ERR_WA1, MD: Water_2nd_hydration_shell, D: Aff_APA_20% (kcal/mol), D: ERR_A20 (kcal/mol), D: Aff_APA_30% (kcal/mol), D: ERR_A30 (kcal/mol), D: Aff_APA_40% (kcal/mol), D: ERR_A40 (kcal/mol), D: Aff_APA (kcal/mol), D: ERR_APA (kcal/mol), D: Aff_DPB_20% (kcal/mol), D: ERR_B20 (kcal/mol), D: Aff_DPB_30% (kcal/mol), D: ERR_B30 (kcal/mol), D: #poses_DPB_40%, D: Aff_DPB_40% (kcal/mol), D: ERR_B40 (kcal/mol), D: Aff_DPB (kcal/mol), D: ERR_DPB (kcal/mol), D: GLN46_B, D: THR89_B, D: ARG128_B, D: VAL177_B, D: GLY179_B, D: SER180_B, D: GLN273_B, D: ASP274_B, D: ARG620_B, QSAR: Bonds, QSAR: Refractivity, QSAR: Hararyindex, QSAR: Plattindex, QSAR: Randicindex, QSAR: Szegedindex, QSAR: Wienerindex, QSAR: Hyperwienerindex, QSAR: Surface (Å^2), QSAR: ASA (Å^2), QSAR: ASAplus (Å^2), QSAR: ASAminus (Å^2), QSAR: Pienergy, QSAR: Isotropic_pol (a.u.), QM: E_thermal (kcal/mol), QM: CV (cal/mol-Kelvin), QM: S (cal/mol-Kelvin)
7	4	D: #poses_DPB_30%, D: LYS134_B, D: PHE136_B, D: GLN176_B, D: PHE178_B, D: ILE277_B, D: PHE615_B, D: PHE617_B
6	6	QSAR: ASA_H (Å^2)
5	2	QM: Rotational_constant_a (GHz), MD: ERR_WA2, MD: RMSF (Å), MD: MPA (Å^2), MD: ERR_MPA (Å^2), MD: ERR_ACY, D: THR130_B, QSAR: Rotatable_bonds, QSAR: Aliphatic_atoms, QSAR: Aliphatic_bonds, QSAR: Chainatoms, QSAR: Chainbonds, P: Δs-SOL (J/mol K), P: Δs-IL-HEAD (J/mol K), P: Δs-IL-GLY (J/mol K), P: Δs-TAILS (J/mol K), P: Δs-LIPID-A (J/mol K), P: Δs-CORE-1 (J/mol K), P: Δs-CORE-2 (J/mol K)
4	12	QSAR: Topolog_surface (Å^2), QSAR: ASA_P (Å^2), QSAR: ASA_H/ASA, QSAR: ASA_P/ASA, P: HB-WATER, P: HB-WATER-IL-HEAD, P: HB-WATER-IL-GLY, P: HB-WATER-CORE-1, P: HB-WATER-CORE-2
3	7	QSAR: Donors, P: HB-IL-HEAD, P: HB-IL-GLY, P: HB-TAILS, P: HB-LIPID-A, P: HB-CORE-1, P: HB-CORE-2
2	3	QSAR: Total_charge (e), QM: Total_dipole_moment (Db), QM: HOMO (Hartree), QM: LUMO (Hartree), P: Δh-SOL (kJ/mol), P: Δh-IL-HEAD, P: Δh-IL-GLY (kJ/mol), P: HB-WATER-TAILS, P: Δh-TAILS (kJ/mol), P: HB-WATER-LIPID-A, P: Δh-LIPID-A (kJ/mol), P: Δh-CORE-1 (kJ/mol), P: Δh-CORE-2 (kJ/mol)
1	8	P: HB-MEM-INTER

Table S1: Ranking number (R#) of individual clusters according to permeation predictability, their cluster number (C#), and the descriptors that belong to each of cluster.

rank	1	2	3	4	5	6	7	8	9	10	11	12	13	14	15	16	17	18	19	20	21
c_j fixed	8	3	4	12	2	6	7	1	36	9	14	32	16	10	24	5	15	19	11	21	34
c_j random	8	3	7	2	12	6	4	1	36	9	13	32	16	10	24	15	19	5	25	11	21

Table S2: Ranking of clusters c_j up to top-21, where we compare the ordering when using a fixed testing sample, with a ranking produced when the testing sample changes randomly on every single iteration. The ordering of the clusters is robust to these changes for the most part.

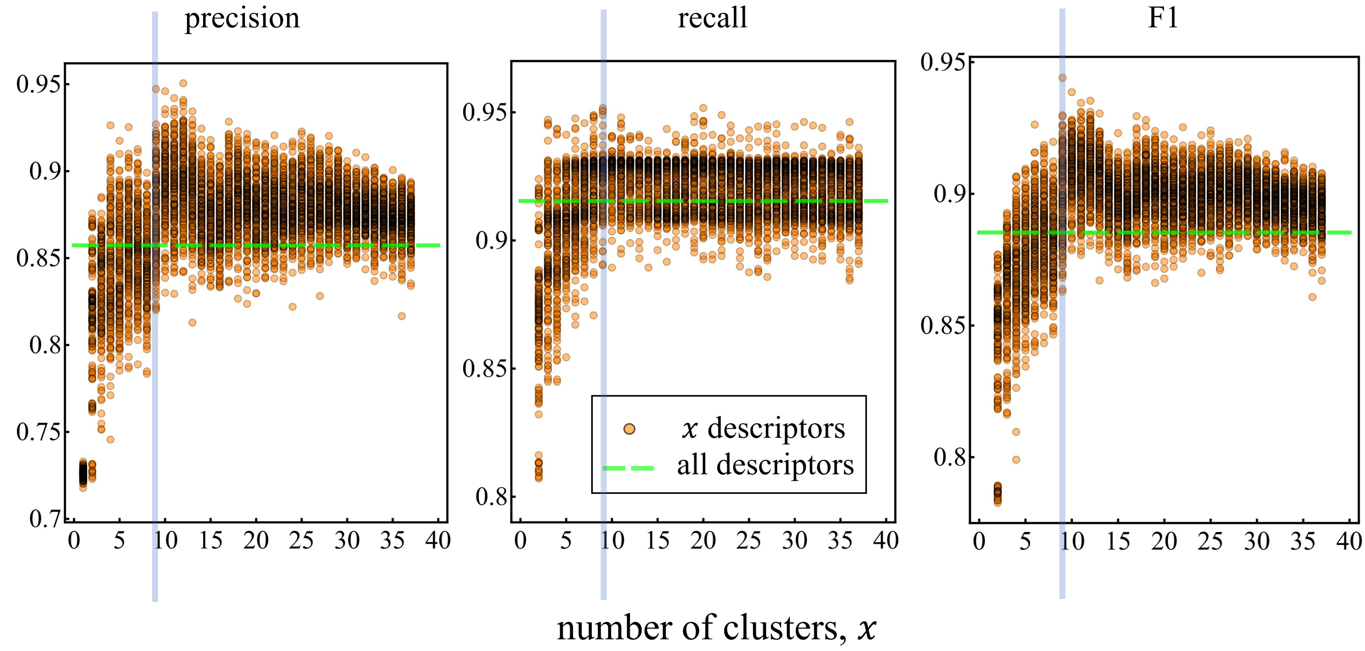

Figure S6: Precision (left), recall (center), and F1 (right) testing results evaluated in the testing portion of the reduction algorithm presented in the main paper. Each orange circle points to the performance of a random combination of x compounds, while the horizontal green line is the evaluation metric when all descriptors are considered. Vertical blue line indicates the $x = 9$ mark.

Evaluation metrics: application We use these definitions into the classification outcome from our model described in Fig. 4 of the main paper, which also shows the result of accuracy. The results for precision, recall, and F1 for each of the tested combination of x descriptors against the testing set of 120 compounds is presented in Fig S6, showing that the cutoff of nine clusters is reasonable in these metrics, as well as, in accuracy.

Figure S7 details the prediction results from the combination listed in Fig. 4c in the main paper. The prediction output, which classifies the testing sample of 120 randomly selected molecules as either strong (class 1) or weak (class 0) permeators, is computed over 50 runs of the classification algorithm, where we do random splits of training/validation of the remaining 480 compounds. From the resultant binary output list of 50 outputs, the average target class of each compound is obtained, which could be interpreted as the probability that a given compound to be a strong permeator (class 1). Indeed, if the average output class is near zero, it indicates the algorithm classifies a given compound is a weak permeator (class 0). A direct comparison between our model's output and the experimental data in the from the IC_{50} ratios is shown in Fig. S7(a), where we see several points of coincidence, as well as, a few misses. Specifically, we see that across the classification of testing compounds as either class 1 or class 0, there are two compounds incorrectly classified as class 1 (FP), and two incorrectly classified as class 0 (FN). Additional model evaluation metrics are presented in Figs. S7(b-c), including the Receiver Operating Characteristic (ROC) curve (solid curve) that is well separated from the limit of random classification (dashed curve). In addition, the evaluation metrics indicates a similar score for recall and for precision, meaning that the algorithm provides a balanced outcome that minimizes almost equally false positives and false negatives. Similarly, the precision or positive predictive value (PPV), and the negative predictive value (NPV), indicates that around 95% of the compounds classified as strong permeators are real, while almost 97% of the compounds classified as weak permeators, are correctly classified as such.

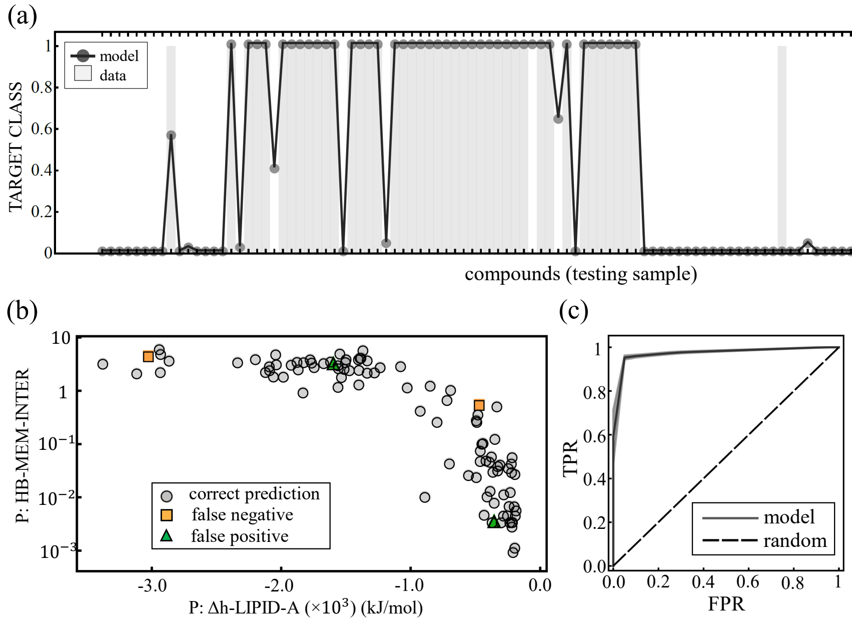

(d)

metric	score
accuracy (a_0)	0.959 ± 0.010
recall	0.940 ± 0.012
precision (PPV)	0.948 ± 0.022
NPV	0.966 ±0.006
specificity	0.028 ± 0.010
class error	0.040 ± 0.010
F1	0.944 ± 0.013

Figure S7: Detailed output of the model in the space of the 120 testing compounds. (a) Direct comparison between the model target class output and the data. The classification output is computed over several random splits of training/validation, hence the average class is reported and could be interpreted as the probability that a given compound is a strong permeator (class 1). (b) Equivalent target class out put but projected in the space of the top-2 descriptors (HB-MEM-INTER, and Δh-LIPID-A). Correct predictions (TP and TN) are shown combined in gray, while the false negatives are orange and the false positive in green. (c) Receiver operating characteristic (ROC) curve associated to our classification model (dark gray) compared to a fully random classification model (black dashed line). (d) Evaluation metrics of the classification model over the testing sample.

1.1.2 Performance of alternative combination of descriptors from the top-9 clusters

Next, we wonder how far, in terms of performance, other combinations in the top-9 clusters identified by our model are from the optimal. From Fig. 4c of the main paper, we see that three of these clusters (6, 8, and 36) have one or two descriptors. This drastically reduces the total number of possible combinations and facilitates a deeper examination of alternative sets of predictors that yield comparable accuracy scores as those presented in Fig. 5 of the main paper. Figure S8 uses the ranking of clusters given by our model (Fig. 4c of the main paper) to inspect the top-3, top-5, and top-9 clusters. For the top-3 [Fig.S8(a)] we look at the model performance of cluster 8 with all combinations of pairs from cluster 3 and 7. The top-5 clusters [Fig.S8(b)] uses the optimal descriptors in cluster 8, 7 and 3, and combine them with all the pairs of clusters 2 and 12. Finally, for the top-9 [S8(c)], we use the optimal values of clusters, 2, 3, 7, 12, 6 and 36, with all the pairs from clusters 1 and 4.

Inspecting the top-3 clusters [Fig.S8(a)] we find that permeation descriptors of each cluster tend to perform higher than QM and QSAR descriptors. More specifically, it is found that the enthalpies of the compounds in different regions of the OM (cluster 3), with the exception of CORE 1, pair well with their hydrogen bond interactions (cluster 7). The top-5 shows that the number of hydrogen bonds in the neighboring of the membrane (HB-WATER) performs well when paired with permeation descriptors associated with entropy beyond the one found by the model (Δs-SOL), which is reasonable given the high correlations found within this quantity when measured across the OM [Fig. S4]. Interestingly, it is also found that the polar surface area (ASA_P) and some combinations of the polar and hydrophobic surface areas to the water accessible surface area (ASA_P/ASA and ASA_H/ASA, respectively) also give a good accuracy score and hence could represent alternative descriptors when the entropies across the OM and the hydrogen bonds in water are not known. Finally, the top-9 shows that, in addition to the docking descriptor found by our model (#poses_DPB_30%), descriptors quantifying the number of contacts between the compound and residues

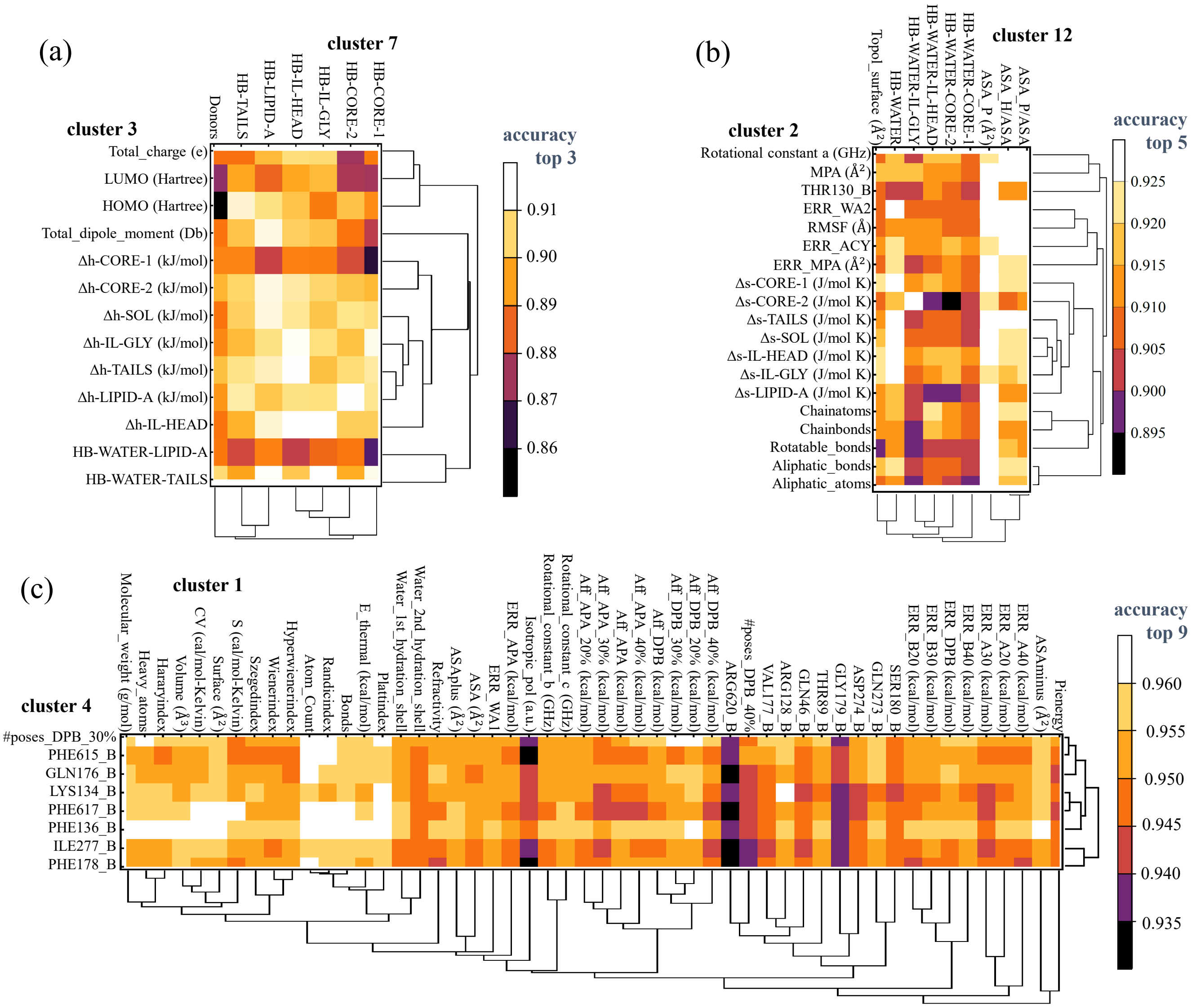

Figure S8: Model performance across clusters. (a) Model's prediction accuracy using all combinations of descriptors from the top-3 clusters (clusters, 8, 3 and 7). (b) Prediction accuracy for all pairs of descriptors from the forth and fifth ranked clusters together with top-3 descriptors from the optimal combination. (c) Prediction accuracy for all pairs of the seventh and eighth ranked clusters alongside with the top-6 descriptors from the optimal set and descriptor ASAplus/ASA (ranked nineth among clusters).

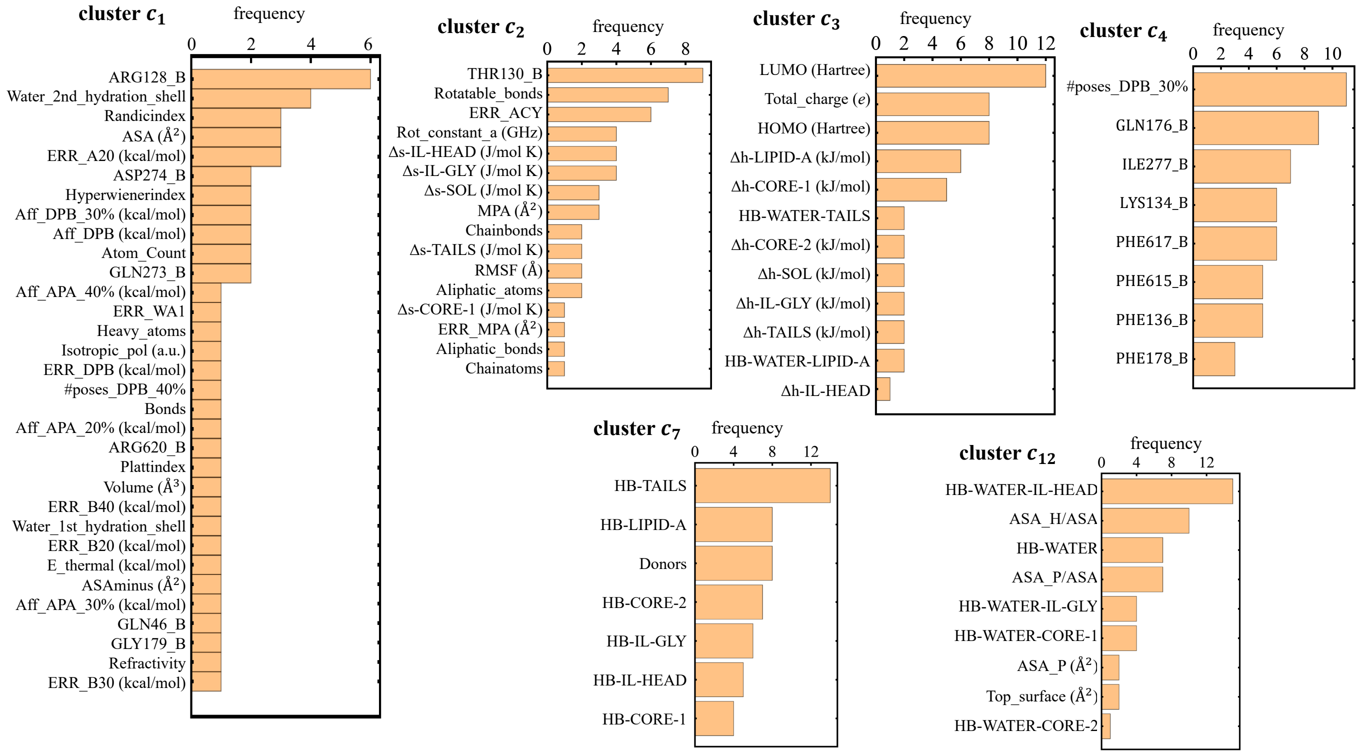

Figure S9: Frequency of identification of specific descriptors for the top-9 clusters across 51 random testing sets of 121 compounds.

PHE136 and PHE617 in the DP of MexB give a good prediction performance when compared to descriptors from cluster 1 associated with the size of the compound beyond the Randic index. Hence, these results, in addition to serve as rigorous test of the outcome of our model, deliver alternative combinations of predictors with comparable performance scores to that of the optimal set found by the model that be helpful when screening candidate drugs.

2 Supplementary Note 2

2.1 High performance permeation prediction from different combinations of descriptors across testing samples

This section explores additional testing samples using the sampling algorithm described in the main paper. For each random testing set of 120 compounds, 200 different combinations of nine descriptors are randomly chosen (one per cluster), trained and validated in the remaining 480 compounds, and the combination of x descriptors that produces the maximum evaluation accuracy in the testing sample is identified. Table S2 lists these descriptors for 50 randomly generated testing samples. A large diversity of combinations is found meaning that the specifics of the data plays a strong role in determining good combinations of descriptors that maximize accuracy.

We further analyze these found combinations by counting the number of times specific descriptors are detected across the different testing samples. The results are shown in the charts of Fig. S9. We find that for the top-9 clusters, there are preferences in choosing some descriptors over other, which highlights their enhanced ability to predict permeation. However, the fact that descriptors are chosen at least once, indicates that for a particular testing sample they play a critical role and therefore its importance cannot be neglected.

cluster c_4	cluster c_3	cluster c_7	cluster c_{12}	cluster c_2	cluster c_1
PHE617_B	HB-W-LIPID-A	HB-CORE-1	HB-W-IL-HEAD	Δs-IL-GLY (J/mol K)	Water_2nd_hydration_shell
GLN176_B	LUMO (Hartree)	HB-TAILS	Topolog_surface (Å^2)	THR130_B	ARG128_B
ILE277_B	HOMO (Hartree)	Donors	ASA_P/ASA	Rotatable_bonds	ERR_B30 (kcal/mol)
#pos_DPB_30%	Δh-TAILS (kJ/mol)	HB-LIPID-A	ASA_H/ASA	Rotatable_bonds	GLN273_B
LYS134_B	Δh-LIPID-A (kJ/mol)	HB-IL-HEAD	Topolog_surface (Å^2)	THR130_B	Refractivity
PHE617_B	Δh-CORE-1 (kJ/mol)	HB-TAILS	HB-W-IL-HEAD	Rotatable_bonds	Water_2nd_hydration_shell
PHE136_B	Total_charge (e)	HB-IL-GLY	HB-W	Δs-IL-HEAD (J/mol K)	GLY179_B
ILE277_B	HOMO (Hartree)	HB-IL-HEAD	ASA_H/ASA	Chainatoms	GLN46_B
#pos_DPB_30%	LUMO (Hartree)	HB-CORE-2	HB-W-IL-HEAD	Rot_constant_a (GHz)	Aff_APA_30% (kcal/mol)
GLN176_B	Δh-LIPID-A (kJ/mol)	HB-TAILS	ASA_H/ASA	Aliphatic_atoms	ARG128_B
PHE615_B	Δh-LIPID-A (kJ/mol)	HB-TAILS	HB-W-IL-HEAD	Δs-IL-HEAD (J/mol K)	ARG128_B
LYS134_B	Total_charge (e)	HB-TAILS	ASA_P (Å^2)	ERR_ACY	Atom_Count
PHE136_B	Δh-IL-GLY (kJ/mol)	HB-CORE-2	HB-W-CORE-1	MPA (Å^2)	ASAminus (Å^2)
PHE617_B	Δh-SOL (kJ/mol)	HB-CORE-2	HB-W-IL-HEAD	ERR_ACY	E_thermal (kcal/mol)
GLN176_B	Total_charge (e)	Donors	ASA_P/ASA	RMSF (A)	ERR_A20 (kcal/mol)
ILE277_B	LUMO (Hartree)	HB-TAILS	HB-W	THR130_B	ARG128_B
LYS134_B	Δh-CORE-2 (kJ/mol)	Donors	HB-W	Rot_constant_a (GHz)	ERR_B20 (kcal/mol)
PHE178_B	Δh-LIPID-A (kJ/mol)	HB-IL-HEAD	HB-W-CORE-1	ERR_ACY	Water_1st_hydration_shell
#pos_DPB_30%	LUMO (Hartree)	Donors	HB-W-IL-GLY	Δs-TAILS (J/mol K)	Atom_Count
PHE136_B	HOMO (Hartree)	HB-LIPID-A	HB-W	Rotatable_bonds	Aff_DPB (kcal/mol)
#pos_DPB_30%	Total_charge (e)	Donors	HB-W-IL-GLY	ERR_ACY	ARG128_B
PHE178_B	HB-W-LIPID-A	HB-LIPID-A	HB-W	THR130_B	GLN273_B
GLN176_B	LUMO (Hartree)	HB-CORE-2	ASA_H/ASA	ERR_ACY	ERR_B40 (kcal/mol)
LYS134_B	Δh-SOL (kJ/mol)	HB-IL-GLY	HB-W-CORE-2	RMSF (A)	Volume (Å^3)
ILE277_B	LUMO (Hartree)	Donors	ASA_P/ASA	THR130_B	ARG128_B
LYS134_B	Δh-TAILS (kJ/mol)	HB-TAILS	HB-W-IL-HEAD	Aliphatic_atoms	Plattindex
ILE277_B	Δh-IL-HEAD	HB-IL-HEAD	HB-W-IL-HEAD	Δs-SOL (J/mol K)	Aff_DPB_30% (kcal/mol)
PHE178_B	Δh-LIPID-A (kJ/mol)	HB-LIPID-A	HB-W	ERR_ACY	ARG620_B
PHE615_B	Δh-CORE-1 (kJ/mol)	HB-IL-GLY	ASA_H/ASA	Aliphatic_bonds	ERR_A20 (kcal/mol)
GLN176_B	HOMO (Hartree)	HB-TAILS	ASA_H/ASA	Rotatable_bonds	Aff_APA_20% (kcal/mol)
LYS134_B	LUMO (Hartree)	Donors	ASA_P/ASA	Δs-SOL (J/mol K)	Randicindex
#pos_DPB_30%	LUMO (Hartree)	HB-LIPID-A	HB-W-IL-HEAD	Rot_constant_a (GHz)	Bonds
PHE615_B	Δh-CORE-1 (kJ/mol)	HB-LIPID-A	ASA_H/ASA	Δs-IL-GLY (J/mol K)	Randicindex
#pos_DPB_30%	Δh-CORE-1 (kJ/mol)	HB-IL-GLY	HB-W-IL-GLY	THR130_B	Aff_DPB (kcal/mol)
#pos_DPB_30%	Total_charge (e)	HB-CORE-2	ASA_H/ASA	MPA (Å^2)	Aff_DPB_30% (kcal/mol)
PHE136_B	LUMO (Hartree)	HB-TAILS	ASA_P/ASA	Rotatable_bonds	ERR_A20 (kcal/mol)
GLN176_B	LUMO (Hartree)	HB-IL-GLY	ASA_P/ASA	Δs-IL-GLY (J/mol K)	Hyperwienerindex
GLN176_B	HOMO (Hartree)	HB-TAILS	ASA_P (Å^2)	Chainbonds	Water_2nd_hydration_shell
PHE617_B	LUMO (Hartree)	HB-CORE-2	HB-W-IL-HEAD	THR130_B	#pos_DPB_40%
PHE617_B	Total_charge (e)	Donors	ASA_H/ASA	MPA (Å^2)	ERR_DPB (kcal/mol)
#pos_DPB_30%	Total_charge (e)	HB-TAILS	HB-W-IL-HEAD	Chainbonds	Isotropic_pol (a.u.)
ILE277_B	Δh-CORE-2 (kJ/mol)	HB-LIPID-A	ASA_P/ASA	Rot_constant_a (GHz)	ASA (Å^2)
GLN176_B	Δh-CORE-1 (kJ/mol)	HB-TAILS	HB-W-IL-HEAD	Δs-IL-HEAD (J/mol K)	ASP274_B
#pos_DPB_30%	HB-W-TAILS	HB-LIPID-A	HB-W-CORE-1	Δs-IL-GLY (J/mol K)	ASP274_B
PHE615_B	Total_charge (e)	HB-TAILS	HB-W-IL-HEAD	ERR_MPA (Å^2)	Heavy_atoms
#pos_DPB_30%	Δh-IL-GLY (kJ/mol)	HB-TAILS	HB-W-IL-HEAD	Rotatable_bonds	ERR_WA1
ILE277_B	HOMO (Hartree)	HB-CORE-1	HB-W-CORE-1	Δs-TAILS (J/mol K)	Water_2nd_hydration_shell
PHE617_B	LUMO (Hartree)	HB-CORE-1	HB-W-IL-HEAD	Δs-CORE-1 (J/mol K)	ASA (Å^2)
PHE136_B	HB-W-TAILS	HB-IL-GLY	HB-W-IL-HEAD	Δs-IL-HEAD (J/mol K)	ASA (Å^2)
GLN176_B	HOMO (Hartree)	HB-IL-HEAD	HB-W-IL-GLY	THR130_B	Hyperwienerindex
PHE615_B	HOMO (Hartree)	HB-CORE-1	ASA_H/ASA	THR130_B	Aff_APA_40% (kcal/mol)
#pos_DPB_30%	Δh-LIPID-A (kJ/mol)	HB-CORE-2	HB-W	Δs-SOL (J/mol K)	Randicindex

Table S3: Example of the variety of combinations of descriptors from the top-9 clusters identified that lead to maximal accuracy for different random testing samples. Since three clusters in the top-9 contain only one (c_8 and c_6) or two descriptors (c_{36}), the table lists those for clusters of size greater than 2 only.

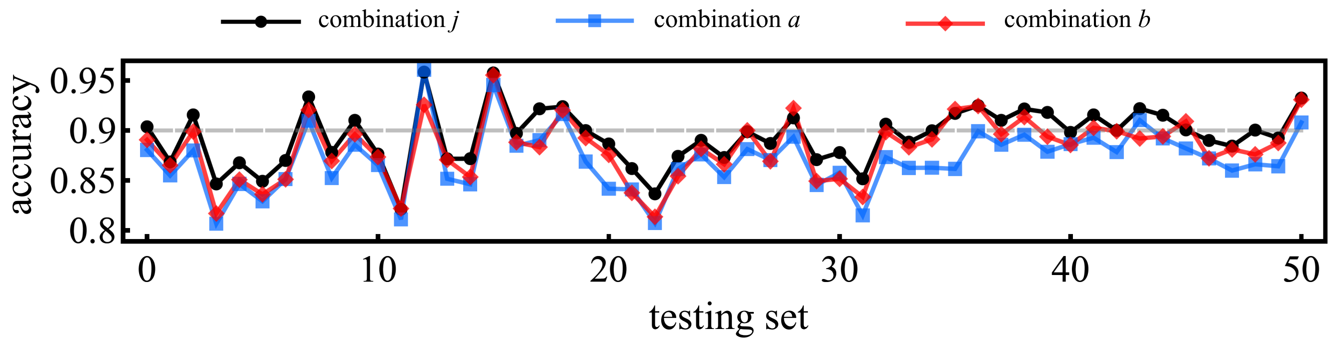

Figure S10: Accuracy score over 50 randomly assembled testing samples using the different combinations of descriptors as explained in the text: combination j, combination a, and combination b. Gray horizontal line indicates the 90% mark in accuracy.

To test this finding even further, we compare the accuracy score in the different testing samples for three types of combinations of descriptors. We call combination j, to the set of descriptors that yield the highest accuracy for the testing set j. Thus, if we are working with 50 testing samples, each of them would have a particular combination of descriptors given by the Table S2. By contrast, we call combination a the set of descriptors that is found for one single testing sample and use it on the different testing samples. For the case shown in Fig. S10, we use the last row of Table S2, which is also the combination listed in Fig. 4c of the main paper. Finally, we use the most frequent descriptor for each cluster as indicated in Fig. S9. This is referred to as combination b. Figure S10 illustrates the results for these three contrasting cases, where we find that the curves tend to follow each other very well and hence the differences between these scores are rather small. Indeed, in most instances, combination a tends to be slightly below the other two cases, but the calculating the average difference with combination j we find that it is only 2.33%, while combination b has an average difference of 1.3% with combination j. Hence, the information provided by the descriptors of the different combinations is highly correlated with the IC_{50} ratios, and it leaves a very small room for improvement or optimization.

3 Supplementary Note 3

3.1 Classification of active compounds according to predictability

The analysis performed in section 2.4 is further examined in order to extract insights about the predictability of the different compounds.

3.1.1 Permeation class threshold based on empirical IC_{50} ratio

The IC_{50} ratio threshold separating the permeation classification of the compounds presented in the main manuscript is 0.5. This choice comes from extensive calculations with several choices of thresholds. For each choice we have run our reduction algorithm over the entire set of clusters of descriptors where we identified the combination that maximizes the classification performance (Fig 4a in the main manuscript). Subsequently, we test these descriptors across 100 randomly constructed testing samples of 120 compounds each where we identify the groups G (always predicted correctly), R (always predicted incorrectly), and B (sometimes predicted correctly and sometimes incorrectly) for each ratio threshold tested (Fig. 6a in the main manuscript). Figure S11 shows that a ratio of 0.5 maximizes the number of compounds predicted correctly (set G on top) at every run, while simultaneously minimizing the number of compounds predicted incorrectly (set R in the middle) and those producing mixed results (set B in the bottom). As shown, other threshold choices produces higher classification errors and larger fluctuations.

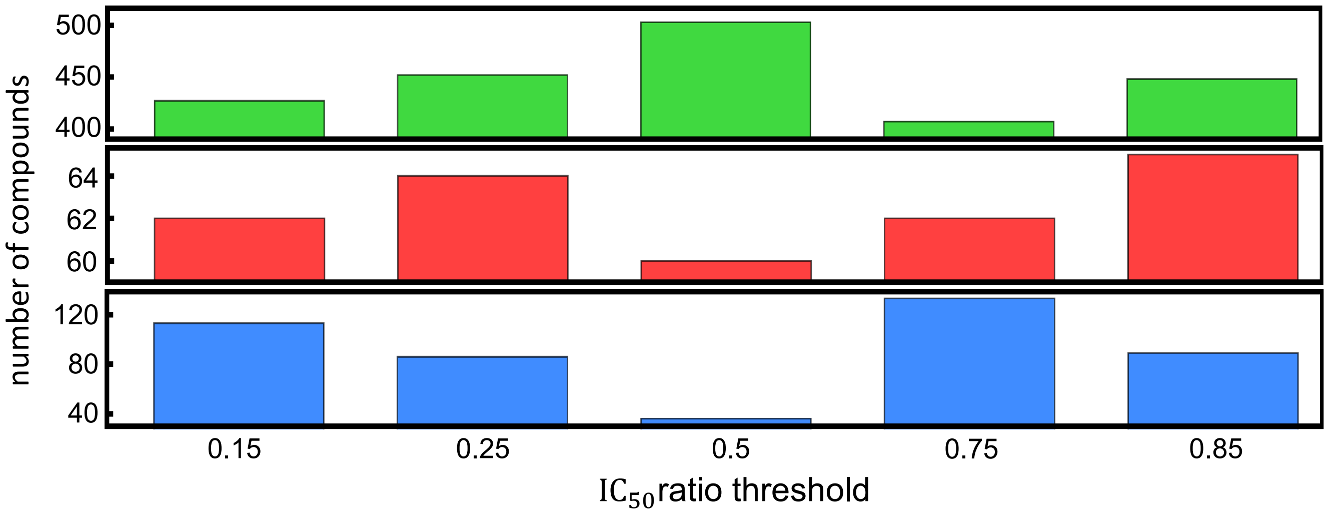

Figure S11: Active compounds classification according to predictability for different choices of IC_{50} ratio threshold. The size of the sets G, R and B, are shown in the top, middle, and bottom panel, respectively. A threshold of 0.5 maximizes the compounds in the set G while minimizing the sets R and B, as shown.

metric/set	all	G + B	G + R	R + B
accuracy	0.868±0.027	0.969±0.016	0.893±0.027	0.720±0.086
recall	0.820±0.051	0.971±0.027	0.843±0.044	0.721±0.132
precision	0.853±0.047	0.951±0.032	0.893±0.042	0.756±0.130
NPV	0.878±0.037	0.981 ±0.017	0.893±0.031	0.698±0.143
specificity	0.097±0.034	0.031±0.021	0.070±0.029	0.266±0.142
class error	0.131±0.027	0.030±0.016	0.106±0.027	0.279±0.086
F1	0.835±0.034	0.960±0.022	0.867±0.035	0.726±0.092

Table S4: Comparison of the different evaluation metrics for different combination of predictability sets: all sets, G+R, G+B, and R+B. For each combination set we show the average metric across 100 randomly assembled testing samples.

3.1.2 Parameter regions associated with the different permeation classes

Focusing on the threshold of 0.5 and for the set of nine descriptors highlighted by the reduction algorithm (see Fig 4 in the main paper), we examine the two-dimensional scatter plots of the compounds of the set G. This yield a total of 36 scatter plots that are shown in Fig. S12. Interestingly, there are clear regions that separate strong (red) and weak (blue) permeators. The set G contain the largest amount of compounds (501), accounting for 83% of all active molecules analyzed. This finding allow us to draw simple rules of permeation based on the descriptor values associated with a given compound. For comparison, Table S4 provides the evaluation metric for different combinations of predictability sets: all, G+B, G+R, and R+B.

In order to compare this behavior of the set G with the compounds of the sets R and B, Fig. S13 illustrates these three sets in the space of the top-2 descriptors that better predict their permeation properties. Similar to the result of the set G, compounds in the set R are also separated into specific regions in the parameter space. However, the regions associated to weak and strong permeators are opposite to those in the compounds of the set G. This explains why they are always missed by the prediction algorithm. Finally, the set B has compounds some of which belong to the pattern shown by the set G and the remaining show the pattern presented in the set R.

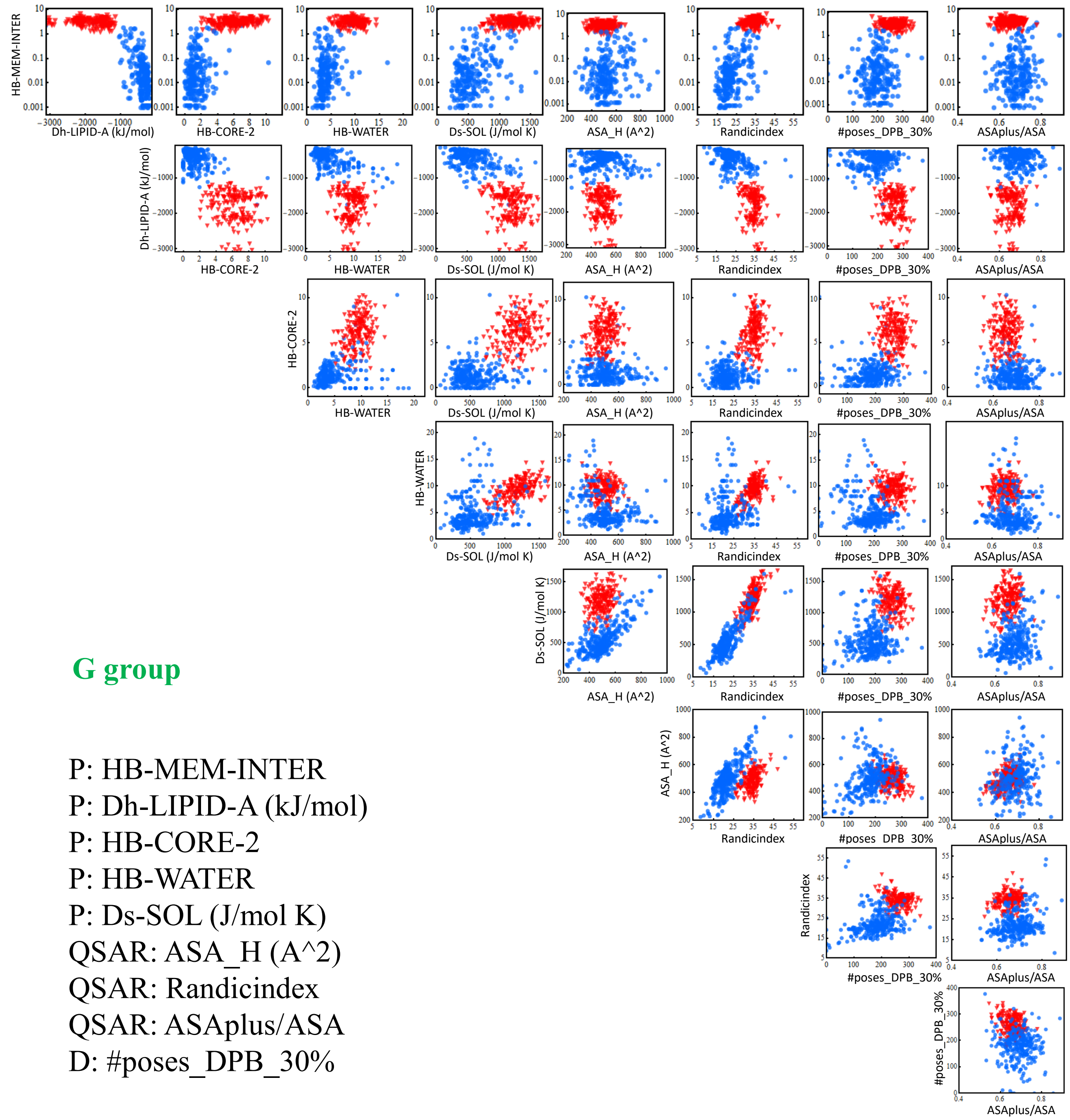

Figure S12: Compounds of the set G projected over the nine descriptors highlighted by the reduction algorithm, as listed in the bottom left. In each of the scatter plots shown, red dots represent strong permeators, while blue are weak permeators. For each row (column) the y-(x-)axis represents a single descriptor, as shown.

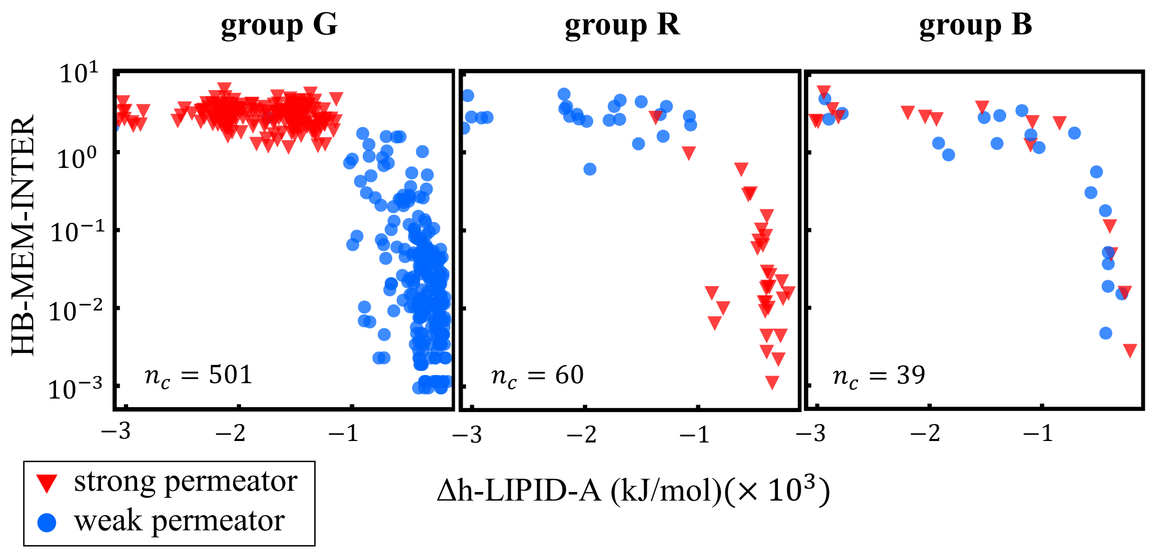

Figure S13: Projection of the 600 active compounds over the top-2 descriptors that predict permeation the best for the three predictive classification groups: set G (left panel), set R (central panel), and set B (right panel).

3.1.3 Structural aspects of compounds in critical subgroups

A classification of the compounds by means of a complete Tanimoto similarity analysis reveals 233 subgroups. As explained in the main manuscript, around 90% of the compounds in sets R and B are concentrated in 10 Tanimoto subgroups. Figure S15 shows the results of our modeling classification in the seven subgroups not shown in Figure 5d of the main manuscript. Our analysis identifies descriptors able to separate weak and strong permeators for some of these subgroups including some compounds of the sets R and B. For other subgroups, however, it remains a challenge. The structural features that distinguish these 10 subgroups are detailed below:

- SB71 are the NSC-33353 analogs made as EPI's. Most of those compounds had a substituted naphthyl amide group present. This is the small subset that didn't have a naphthyl amide but instead had phenyl or biphenyl. So, this seems clear relative to the other compounds in the NSC-33353 series. What is also interesting, it is these phenyl and biphenyl are currently being made because they work in WT Acinetobacter. But not E. coli or PAO1.

- SB117 is full of NSC-60339 analogs. Unlike the subgroup below, these typically have 5-aromatic rings in them or more and tend to be more symmetrical with 2 or more of the dihydroimidazoline groups. So, this subgroup is mostly the original hits and analogs. The subgroup below (SB118) is typically 2-3-aromatic and non-symmetrical with only one dihydroimiazoline group. So, that's the biggest difference. This subgroup is the analogs with more than one dihydroimidazoline moiety in them. They are going to have larger MW compared to SB118 as well.

- SB118 is composed of the NSC-60339 analogs we got from the NCI and synthesized to generate new EPI's. They all have that same group (4-(4,5-dihydro-1H-imidazol-2-yl)aniline) that all of the NSC-60339 analogs have in it. These are generally fairly linear with only a few rotatable bonds and generally fairly lipophilic.

- SB112 is a mix of the original NSC-125028 compounds (MUKB inhibitors) and NSC-60339 (Original EPI compounds). There is a mix of charged and uncharged compounds. Finally, analogs and the intermediates to make them. They are all polyaromatic with amide linkers. There are not many rotatable bonds, generally lipophilic.

- SB167 is an amide derived from 3-aminoquinoline with one exception is cmpd, which is not a quinoline

but a naphthyl. It could have been in this group or in SB168. The reason it was not could be that most, but not all, of SB167 also has a formal +1 charge while most of SB168 does not.

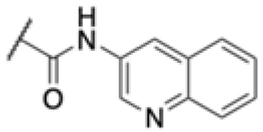

Figure S14: Common structural motif (3-aminoquinoline) that is present in all compounds of this subgroup, which could help identify these compounds and prevent errors in their classification

- SB168 all have an amide derived from a 6-aminoquinoline group present in their structure. There were 3 exceptions. One of them has a 6-substituted quinoline in it, but it is not an amide. And then two compounds which do not have the quinoline but instead have a cyclic boronate derived amide in them.
- SB169 has the phenyl ethyl side chain (from the unnatural amino acid) as opposed to the phenyl alanine or substituted phenyl alanine, that is present in most of the others.
- 12/13 compounds of SB170 contain a phenyl boronic acid group. In general, these are less rigid and lower MW than the previous Rempex subgroups discussed. The one non-boronic acid has a phenol instead but otherwise does look somewhat similar and the PKA's of phenol and boronic acid are somewhat close. This subgroup is somewhat related structurally to SB223. More so than the other Rempex subgroups.
- SB201 are benzothiazoles (BTZs) from JKW lab. The majority of the BTZ's made had a n-propylimidazole group on them. That was key for the antibacterial activity but also made them substrates for efflux and contributed to poor PK. A handful of them were made without the n-propylimidazole group to try and change the properties and hopefully improve their permeability etc. That small subset is captured in subgroup SB201. the n-propylimidazole group was replaced by simple heterocyclic groups such as piperidine, pyrrolidine etc.
- SB223 are Rempex compounds. With one exception none of these compounds are charged (N-methylated). There is also one boronic acid in this group. They are also more in general have more SP3 character. MW is probably lower on average compared to the other Rempex subgroups, but certainly less rigid, not as elaborated structurally and not formally charged.

4 Supplementary Note 4

4.1 Descriptor ranges associated to weak and strong permeation

The consistency of the patterns found for the largest group of compounds (set G), allows us to draw simple rules of permeation based on a compound's parameter values. To this end, we use the top-9 clusters that better correlate with the IC_{50} ratios. This results in a total of 112 descriptors. Tables S5 and S6 shows the ranking of these descriptors that better correlate with permeation individually. In these tables we compare the metrics for the set G with that of all of the active compounds. The ranking is according to accuracy in the latter set. The value t_c is the critical threshold dividing strong and weak permeators according to column L|R. For example, when looking at descriptor δ_j, if $\delta_j > t_c$, its classification would be that of R (i.e., W or S for weak and strong permeator, as listed in the L|R column), and it is L, otherwise. The threshold is estimated using a Support Vector Machine [38] (SVM) algorithm with linear kernel that separates the strong and weak permeators data using a maximum-margin hyperplane. For the one-dimensional case (i.e., one descriptor), t_c is a single point. Extensions to two and three dimensions are also implemented for comparison, where the SVM find the optimal line [Fig. S12] or plane [Fig. S13] to separate the target classes, respectively.

					set G			all compounds		
r	c_j	descriptor	t_c	L|R	PPV	NPV	a_0	PPV	NPV	a_0
1	8	P: HB-MEM-INTER	1.361	W|S	0.955	0.99	0.976	0.836	0.883	0.864
2	3	P: Δh-LIPID-A (kJ/mol)	-988.85	S|W	0.937	1.	0.974	0.816	0.891	0.859
3	3	P: Δh-TAILS (kJ/mol)	-1011.43	S|W	0.923	0.997	0.966	0.806	0.89	0.854
4	3	P: Δh-SOL (kJ/mol)	-979.863	S|W	0.927	0.987	0.962	0.81	0.88	0.851
5	3	P: Δh-IL-GLY (kJ/mol)	-1010.67	S|W	0.931	0.983	0.962	0.81	0.88	0.851
6	3	P: Δh-IL-HEAD	-952.17	S|W	0.921	0.977	0.954	0.804	0.873	0.844
7	2	QSAR: Rotatable_bonds	8.374	W|S	0.885	0.993	0.946	0.781	0.892	0.842
8	7	P: HB-CORE-2	3.343	W|S	0.91	0.964	0.942	0.796	0.876	0.842
9	7	P: HB-IL-HEAD	3.342	W|S	0.897	0.99	0.95	0.78	0.89	0.841
10	2	D: THR130_B	26.007	W|S	0.877	0.993	0.942	0.777	0.889	0.839
11	12	QSAR: ASA_P (Å^2)	184.089	W|S	0.865	0.996	0.938	0.788	0.878	0.839
12	2	QSAR: Chainatoms	15.428	W|S	0.892	0.986	0.946	0.782	0.88	0.837
13	1	D: THR89_B	49.831	W|S	0.903	0.973	0.944	0.784	0.875	0.836
14	7	P: HB-IL-GLY	3.121	W|S	0.907	0.973	0.946	0.788	0.871	0.836
15	1	D: GLN46_B	115.409	W|S	0.869	0.996	0.94	0.764	0.893	0.834
16	1	D: #poses_DPB_40%	104.373	W|S	0.862	1.	0.938	0.76	0.895	0.833
17	3	P: Δh-CORE-2 (kJ/mol)	-1141.99	S|W	0.869	0.996	0.94	0.768	0.886	0.833
18	2	QSAR: Chainbonds	17.124	W|S	0.879	0.983	0.938	0.773	0.876	0.831
19	3	P: HB-WATER-TAILS	3.154	W|S	0.866	0.976	0.929	0.771	0.878	0.831
20	3	QM: Total_dipole_moment (Db)	32.652	W|S	0.896	0.957	0.933	0.782	0.86	0.828
21	4	D: GLN176_B	131.529	W|S	0.825	0.996	0.917	0.735	0.889	0.816
22	1	D: VAL177_B	74.43	W|S	0.81	0.993	0.907	0.725	0.888	0.809
23	7	P: HB-CORE-1	2.857	W|S	0.847	0.942	0.903	0.751	0.854	0.809
24	1	D: ARG620_B	113.094	W|S	0.801	0.996	0.903	0.718	0.895	0.808
25	3	P: Δh-CORE-1 (kJ/mol)	-1089.79	S|W	0.81	0.993	0.907	0.724	0.885	0.808
26	2	MD: ERR_WA2	5.654	W|S	0.837	0.952	0.903	0.745	0.853	0.806
27	3	QSAR: Total_charge (e)	1.393	W|S	0.795	1.	0.901	0.716	0.895	0.806
28	1	QM: E_thermal (kcal/mol)	335.469	W|S	0.797	0.992	0.899	0.716	0.889	0.804
29	12	QSAR: ASA_H/ASA	0.743	S|W	0.785	0.996	0.893	0.724	0.877	0.804
30	12	QSAR: ASA_P/ASA	0.257	W|S	0.785	0.996	0.893	0.724	0.877	0.804
31	2	P: Δs-SOL (J/mol K)	808.542	W|S	0.807	0.978	0.899	0.72	0.874	0.801
32	1	D: ARG128_B	79.772	W|S	0.776	0.988	0.885	0.705	0.89	0.798
33	1	QSAR: Randicindex	27.08	W|S	0.788	0.985	0.891	0.707	0.882	0.796
34	1	QSAR: Pienergy	53.92	W|S	0.799	0.96	0.887	0.732	0.846	0.796
35	3	QM: LUMO (Hartree)	-0.201	S|W	0.763	0.996	0.879	0.695	0.906	0.796
36	3	P: HB-WATER-LIPID-A	3.035	W|S	0.8	0.978	0.895	0.713	0.873	0.796
37	1	QSAR: Atom_Count	61.33	W|S	0.785	0.985	0.889	0.705	0.882	0.794
38	1	QSAR: Surface (Å^2)	657.716	W|S	0.788	0.981	0.889	0.706	0.879	0.794
39	2	MD: MPA (Å^2)	61.503	W|S	0.779	1.	0.891	0.701	0.89	0.794
40	2	P: Δs-CORE-2 (J/mol K)	553.616	W|S	0.791	0.97	0.887	0.712	0.87	0.794
41	4	D: PHE615_B	159.34	W|S	0.771	0.992	0.883	0.698	0.889	0.793
42	1	QSAR: Plattindex	221.664	W|S	0.776	0.988	0.885	0.699	0.884	0.791
43	1	QM: CV (cal/mol K)	119.661	W|S	0.781	0.981	0.885	0.701	0.879	0.791
44	7	QSAR: Donors	3.885	W|S	0.755	1.	0.875	0.689	0.905	0.791
45	12	QSAR: Topological_surface (Å^2)	119.181	W|S	0.799	0.974	0.893	0.711	0.862	0.791
46	1	QSAR: Bonds	63.899	W|S	0.772	0.984	0.881	0.696	0.883	0.789
47	2	P: Δs-CORE-1 (J/mol K)	446.791	W|S	0.803	0.964	0.891	0.712	0.857	0.789
48	2	P: Δs-IL-GLY (J/mol K)	647.777	W|S	0.779	0.973	0.881	0.701	0.871	0.788
49	1	QSAR: Volume (Å^3)	418.747	W|S	0.771	0.981	0.879	0.694	0.877	0.786
50	1	QM: S (cal/mol K)	209.437	W|S	0.765	0.992	0.879	0.691	0.885	0.786
51	2	MD: RMSF (Å)	0.196	W|S	0.776	0.985	0.883	0.691	0.869	0.781
52	1	MD: Water_1st_hydration_shell	55.394	W|S	0.756	0.98	0.869	0.684	0.878	0.779
53	1	QSAR: Hararyindex	136.77	W|S	0.77	0.962	0.871	0.692	0.864	0.779
54	2	P: Δs-LIPID-A (J/mol K)	551.215	W|S	0.773	0.962	0.873	0.696	0.857	0.779
55	4	D: LYS134_B	116.579	W|S	0.758	0.977	0.869	0.685	0.876	0.779
56	1	MD: Water_2nd_hydration_shell	114.493	W|S	0.753	0.98	0.867	0.682	0.878	0.778
57	1	D: ASP274_B	64.911	W|S	0.734	0.984	0.855	0.677	0.889	0.778
58	1	QSAR: Refractivity	144.05	W|S	0.748	0.972	0.861	0.683	0.875	0.778
59	2	P: Δs-IL-HEAD (J/mol K)	703.76	W|S	0.762	0.969	0.869	0.688	0.866	0.778

Table S5: Individual descriptor ranges associated with strong (SP) and weak (WP) OM permeation for the top-60 descriptors according to the accuracy score of all active compounds. For each ranking descriptor r belonging to cluster c_j, the threshold t_c separating the target classes, is listed in the measurement units of the descriptor. Column L|R indicates whether WP are associated with descriptor values smaller than t_c and hence SP associated with values larger than t_c (entry W|S), or vice versa (entry S|W). The evaluation metrics of positive predictive value (PPV), negative predictive value (NPV) and accuracy (a_0) are listed for each descriptor for the compounds of the set G only and for all active compounds, as indicated.

					set G			all compounds		
r	c_j	descriptor	t_c	L|R	PPV	NPV	a_0	PPV	NPV	a_0
60	1	QSAR: Heavy_atoms	33.457	W|S	0.764	0.966	0.869	0.687	0.863	0.776
61	1	MD: ERR_WA1	4.355	W|S	0.771	0.941	0.863	0.696	0.847	0.776
62	1	QSAR: Wienerindex	4272.3	W|S	0.777	0.942	0.867	0.696	0.847	0.776
63	1	D: SER180_B	52.181	W|S	0.749	0.965	0.859	0.682	0.867	0.774
64	1	QSAR: Hyperwienerindex	23670.	W|S	0.788	0.92	0.863	0.704	0.832	0.774
65	1	QSAR: Molecular_weight (g/mol)	473.818	W|S	0.755	0.965	0.863	0.681	0.864	0.773
66	2	MD: ERR_MPA (Å^2)	5.67	W|S	0.749	0.976	0.863	0.679	0.869	0.773
67	2	MD: ERR_ACY	0.396	W|S	0.758	0.977	0.869	0.68	0.867	0.773
68	2	QSAR: Aliphatic_atoms	19.48	W|S	0.748	0.984	0.865	0.676	0.874	0.773
69	12	P: HB-WATER-IL-GLY	3.726	W|S	0.761	0.941	0.857	0.693	0.844	0.773
70	1	D: GLN273_B	39.855	W|S	0.743	0.976	0.859	0.678	0.866	0.771
71	1	D: ERR_A30 (kcal/mol)	0.603	W|S	0.805	0.891	0.857	0.719	0.801	0.768
72	2	P: Δs-TAILS (J/mol K)	601.65	W|S	0.758	0.955	0.861	0.682	0.849	0.768
73	12	P: HB-WATER-CORE-1	3.511	W|S	0.771	0.931	0.859	0.695	0.828	0.768
74	1	D: ERR_APA (kcal/mol)	0.6	W|S	0.77	0.906	0.847	0.7	0.818	0.766
75	12	P: HB-WATER	6.742	W|S	0.733	0.949	0.843	0.67	0.851	0.761
76	1	QSAR: ASA (Å^2)	684.798	W|S	0.742	0.939	0.845	0.67	0.841	0.758
77	3	QM: HOMO (Hartree)	-0.305	S|W	0.698	0.991	0.831	0.644	0.914	0.758
78	1	D: ERR_A20 (kcal/mol)	0.627	W|S	0.763	0.905	0.843	0.685	0.813	0.756
79	7	P: HB-TAILS	2.122	W|S	0.767	0.888	0.837	0.691	0.805	0.756
80	1	D: ERR_A40 (kcal/mol)	0.556	W|S	0.721	0.938	0.831	0.664	0.843	0.755
81	2	QSAR: Aliphatic_bonds	21.404	W|S	0.716	0.979	0.841	0.654	0.867	0.755
82	1	D: ERR_DPB (kcal/mol)	0.735	W|S	0.757	0.89	0.833	0.685	0.802	0.751
83	1	D: GLY179_B	73.627	W|S	0.709	0.955	0.829	0.649	0.868	0.751
84	1	QSAR: Szegedindex	5787.5	W|S	0.743	0.916	0.837	0.671	0.82	0.751
85	7	P: HB-LIPID-A	1.825	W|S	0.777	0.877	0.837	0.686	0.796	0.75
86	1	QSAR: ASAminus (Å^2)	221.86	W|S	0.726	0.928	0.831	0.662	0.823	0.746
87	1	D: ERR_B40 (kcal/mol)	0.733	W|S	0.724	0.895	0.817	0.658	0.806	0.738
88	4	D: #poses_DPB_30%	214.971	W|S	0.692	0.958	0.817	0.635	0.859	0.738
89	1	D: ERR_B20 (kcal/mol)	0.726	W|S	0.721	0.879	0.81	0.661	0.798	0.736
90	1	D: Aff_APA_30% (kcal/mol)	-9.719	S|W	0.697	0.958	0.821	0.635	0.846	0.735
91	1	D: Aff_DPB_20% (kcal/mol)	-10.321	S|W	0.68	0.978	0.813	0.629	0.866	0.735
92	1	D: Aff_DPB_40% (kcal/mol)	-10.393	S|W	0.674	0.982	0.81	0.625	0.871	0.733
93	1	D: Aff_DPB_30% (kcal/mol)	-10.361	S|W	0.674	0.982	0.81	0.624	0.868	0.731
94	12	P: HB-WATER-IL-HEAD	4.076	W|S	0.699	0.898	0.804	0.648	0.806	0.731
95	1	D: Aff_APA (kcal/mol)	-9.447	S|W	0.663	0.952	0.794	0.625	0.851	0.728
96	1	D: ERR_B30 (kcal/mol)	0.737	W|S	0.698	0.888	0.8	0.644	0.803	0.728
97	4	D: PHE617_B	95.925	W|S	0.681	0.93	0.802	0.631	0.832	0.728
98	1	D: Aff_APA_40% (kcal/mol)	-9.665	S|W	0.665	0.977	0.802	0.619	0.866	0.726
99	1	D: Aff_DPB (kcal/mol)	-10.28	S|W	0.667	0.982	0.804	0.618	0.869	0.726
100	1	D: Aff_APA_20% (kcal/mol)	-9.634	S|W	0.663	0.944	0.792	0.616	0.846	0.72
101	1	QSAR: ASAplus (Å^2)	462.964	W|S	0.678	0.892	0.788	0.619	0.799	0.71
102	4	D: PHE136_B	123.694	W|S	0.632	0.944	0.766	0.591	0.837	0.697
103	2	QM: Rotational_constant_a (GHz)	0.324	S|W	0.614	1.	0.758	0.577	0.876	0.688
104	12	P: HB-WATER-CORE-2	3.906	W|S	0.65	0.844	0.754	0.604	0.756	0.687
105	1	QM: Isotropic_pol (a.u.)	406.648	W|S	0.634	0.827	0.738	0.592	0.759	0.68
106	4	D: ILE277_B	123.369	W|S	0.605	0.855	0.724	0.58	0.783	0.677
107	1	QM: Rotational_constant_c (GHz)	0.053	S|W	0.586	0.967	0.724	0.555	0.863	0.663
108	4	D: PHE178_B	176.99	W|S	0.575	0.863	0.7	0.558	0.784	0.657
109	1	QM: Rotational_constant_b (GHz)	0.066	S|W	0.533	0.948	0.661	0.52	0.86	0.62
110	36	QSAR: ASAplus/ASA	0.677	S|W	0.5	0.742	0.615	0.502	0.699	0.594
111	36	QSAR: ASAminus/ASA	0.323	W|S	0.5	0.742	0.615	0.502	0.699	0.594
112	6	QSAR: ASA_H (Å^2)	500.76	S|W	0.391	0.622	0.5	0.423	0.608	0.512

Table S6: Individual descriptor ranges associated with strong (SP) and weak (WP) OM permeation for the descriptors below the top-60 according to the accuracy score of all active compounds. For each ranking descriptor r belonging to cluster c_j, the threshold t_c separating the target classes, is listed in the measurement units of the descriptor. Column L|R indicates whether WP are associated with descriptor values smaller than t_c and hence SP associated with values larger than t_c (entry W|S), or vice versa (entry S|W). The evaluation metrics of positive predictive value (PPV), negative predictive value (NPV) and accuracy (a_0) are listed for each descriptor for the compounds of the set G only and for all active compounds, as indicated.

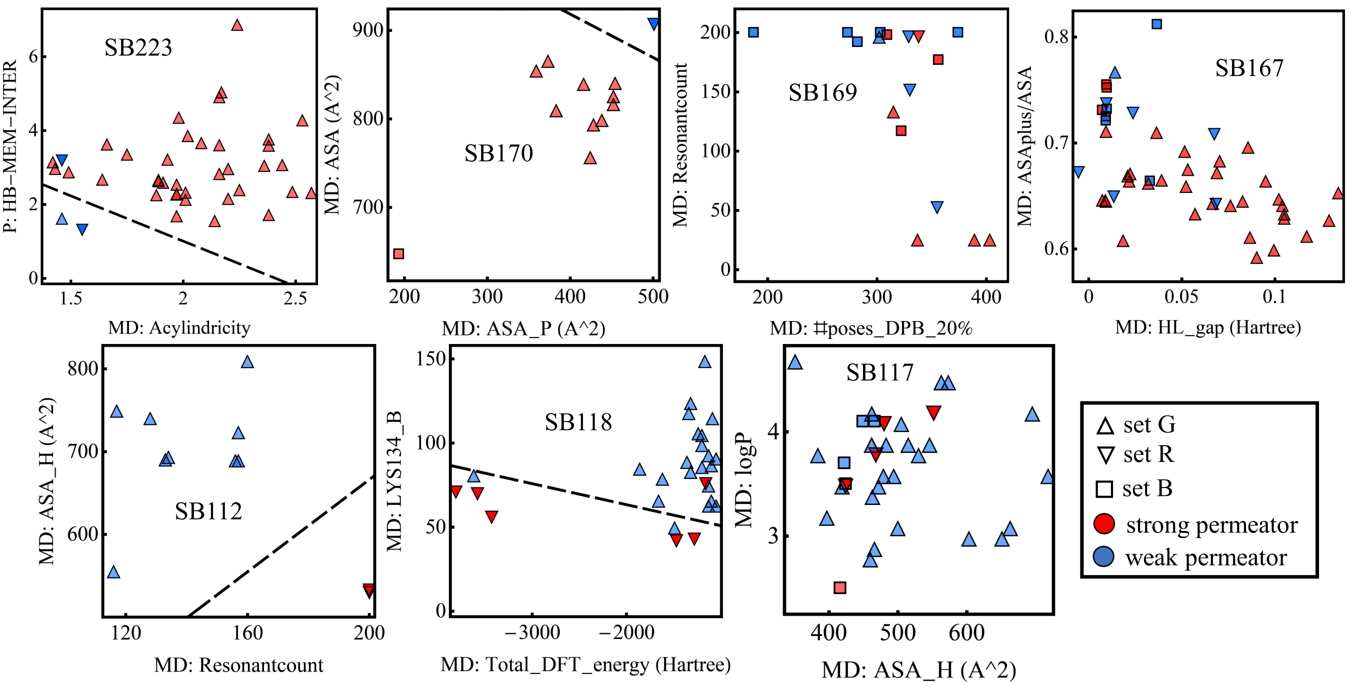

Figure S15: Complementary analysis of relevant descriptors for the remaining seven Tanimoto subgroups that contain relevant number of compound of sets R and B, as illustrated in Figure 5d of the main manuscript. In cases where the separation between weak and strong permeators is somewhat clear, it is marked by a class boundary black line found using an SVM algorithm.

A similar analysis now using sets of two- and three-descriptors is also performed, where we trained a 2- and 3-dimensional support vector machine on all combinations of two and three descriptors of the top-9 clusters (no more than one descriptor per cluster per combination), respectively. Accordingly, we found an enhancement in the performance accuracy for different combinations of two and three descriptors. For simplicity, we have use a straight line and a plane for the cut off of the two- and three-descriptor analysis, respectively. The top-40 combinations and corresponding parameters are listed in the Tables S7 and S8, while we highlight four sets of three-descriptors combinations that classifies well strong and weak permeators (see Fig. S17).

The two-descriptor analysis highlights the role of the electrostatic properties and the electronic structure, together with, the hydrophobic and polar area, of the compounds [Fig. S16]. These pairs of descriptors yield accuracy scores up to 88.1% computed across the entire set of active compounds. In addition, we find several pairs of descriptors comprised by one of permeation (either HB or enthalpy) and one of docking (#poses or contacts to key residues in DP of MexB), or permeation with QM or QSAR descriptors. Finally, the three-descriptor analysis finds the QSAR descriptor quantifying the ratio of the hydrophobic area to the water accessible surface area (i.e., ASA_H/ASA) in myriad combinations with either one permeation and one docking descriptor, or one docking with another QSAR or QM. This separation talks about an interesting relationship among the different types of descriptors and how their information can be harnessed in order to better identify strong and weak permeators. Some examples are found in Fig. S17 reaching accuracy scores of 88.42% obtained across the whole set of active compounds.

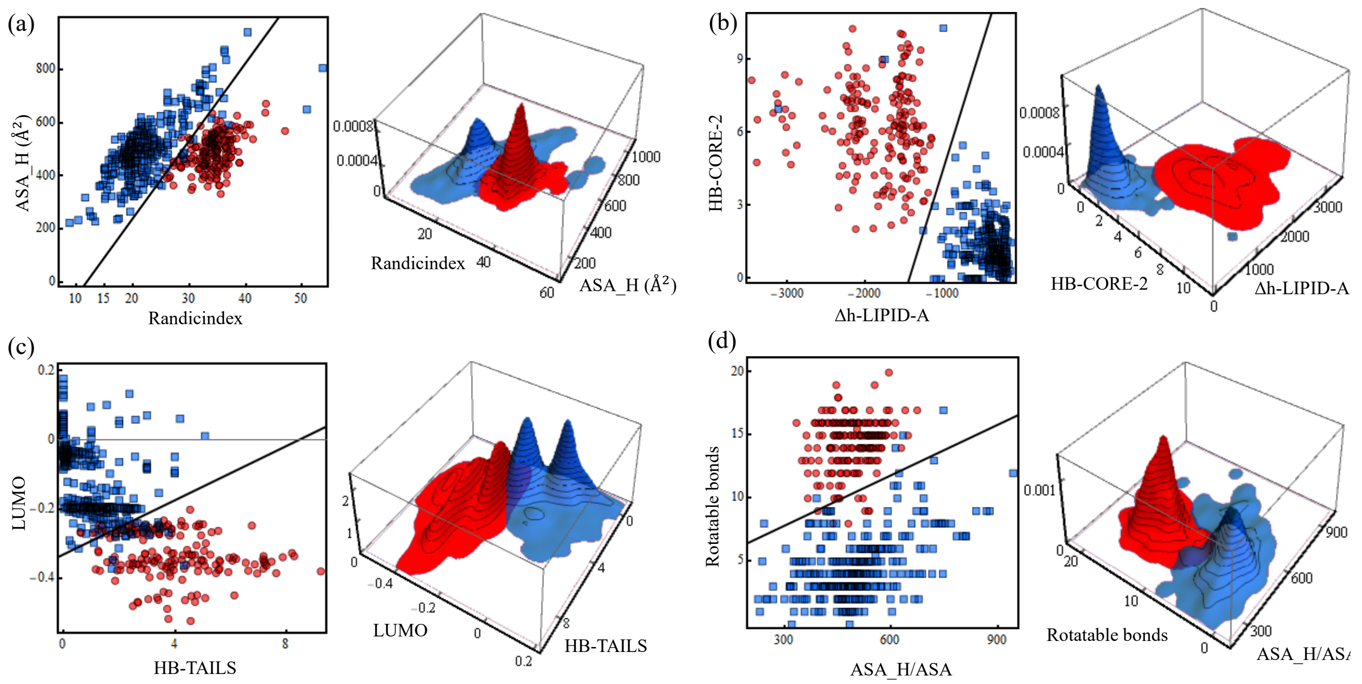

Figure S16: 2-dimensional analysis of the parameter regions associated to weak (blue) and strong (red) permeation for four sets of highly accurate predictors found. Scatter plot at the left of each panel is equivalent to the density distribution plots at the right. Active compounds of the set G are shown on each panel. (a) Randicindex (QSAR), ASA_H (Å^2) (QSAR). (b) Δh-LIPID-A (permeation), HB-CORE-2 (permeation). (c) LUMO (QM), HB-TAILS (permeation). (d) Rotatable bonds (QSAR), ASA_H/ASA (QSAR). Straight line in the left panel of each figure results from the analysis done by the two-dimensional SVM algorithm. The specifics of the function is given in Table S7.

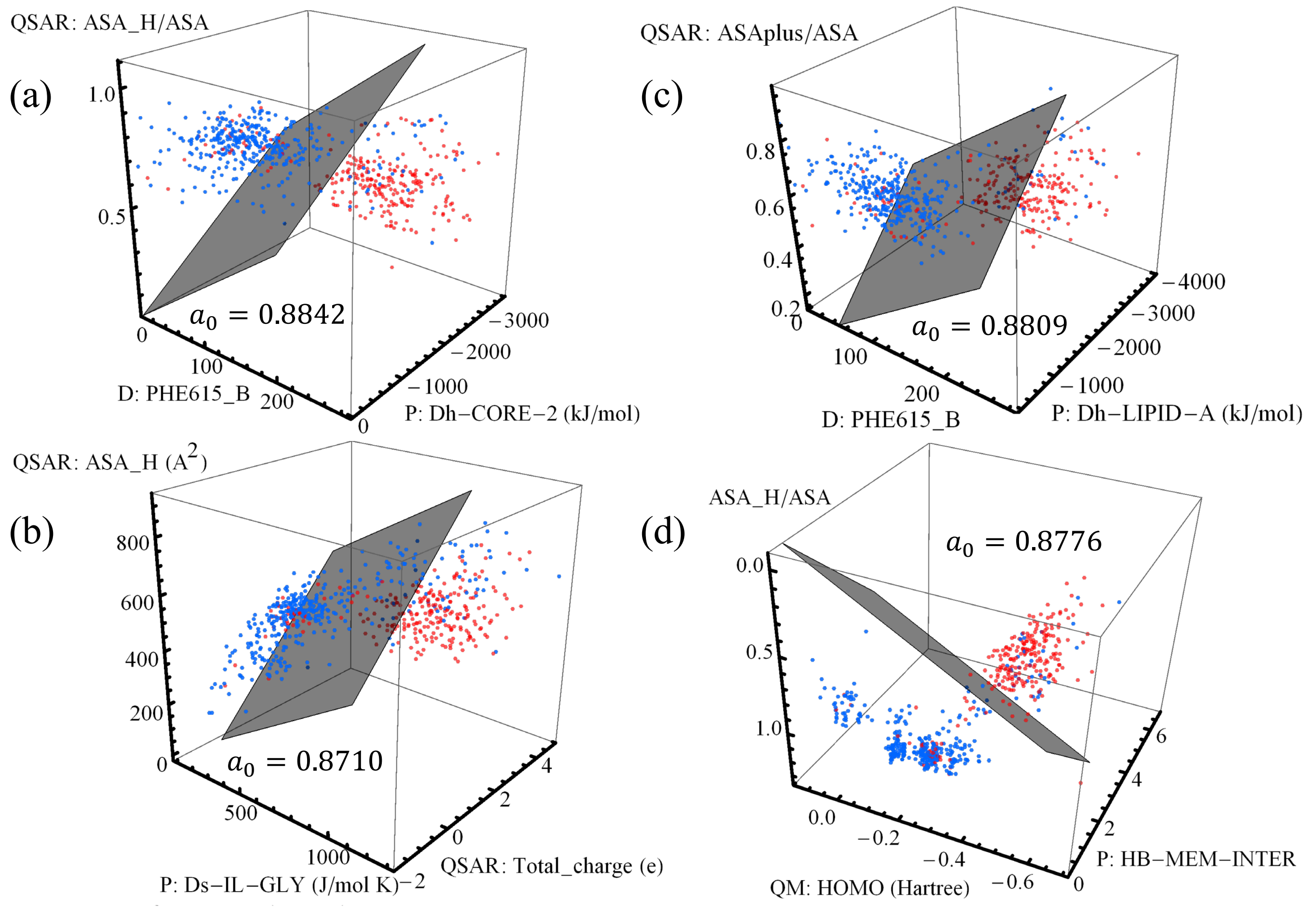

Figure S17: 3-dimensional analysis of the parameter regions associated to weak (blue) and strong (red) permeation for four sets of highly accurate predictors found. All 605 active compounds are shown on each panel. (a) PHE615_B (docking), Δh-CORE-2 (permeation), ASA_H/ASA (QSAR). (b) Δs-IL-GLY (permeation), Total charge (QSAR), ASA_H(Å^2)(QSAR). (c) PHE615_B (docking), Δh-LIPID-A (permeation), ASAplus/ASA (QSAR). (d) HOMO (QM), HB-MEM-INTER (permeation), ASA_H/ASA (QSAR). Accuracy score a_0 for each panel is also shown. Gray plane results from the analysis done by the three-dimensional SVM algorithm. The specifics of the function is given in Table S8.

								set G			all compounds		
r	c_i	c_j	descriptor i	descriptor j	a	b	L|R	PPV	NPV	a_0	PPV	NPV	a_0
1	3	12	QSAR: Total_charge (e)	QSAR: ASA_H/ASA	0.105	0.533	W|S	0.9848	1	0.9941	0.8571	0.8972	0.881
2	3	12	QSAR: Total_charge (e)	QSAR: ASA_P (Å^2)	-46.782	286.671	W|S	0.9847	0.9935	0.9901	0.8589	0.8901	0.8777
3	3	12	QM: LUMO (Hartree)	QSAR: ASA_H/ASA	-1.03	0.464	S|W	0.9848	0.9968	0.9921	0.8619	0.888	0.8777
4	3	12	QM: LUMO (Hartree)	QSAR: ASA_P/ASA	0.928	0.506	S|W	0.9798	0.9968	0.9901	0.8589	0.8901	0.8777
5	3	12	QSAR: Total_charge (e)	QSAR: ASA_P/ASA	-0.094	0.437	W|S	0.9701	1	0.9881	0.8468	0.8964	0.876
6	3	4	P: Δh-LIPID-A (kJ/mol)	D: PHE615_B	0.105	290.942	S|W	0.9799	1	0.9921	0.8496	0.8942	0.876
7	4	12	D: PHE615_B	QSAR: ASA_P (Å^2)	-1.806	520.791	W|S	0.9846	0.9904	0.9881	0.8613	0.8856	0.876
8	1	3	D: #poses_DPB_40%	P: Δh-TAILS (kJ/mol)	11.001	-2569.72	W|S	0.9799	1	0.9921	0.8462	0.8939	0.8744
9	1	12	D: #poses_DPB_40%	QSAR: ASA_P (Å^2)	-2.495	531.439	W|S	0.9897	0.9936	0.9921	0.8577	0.8852	0.8744
10	1	3	D: GLN46_B	P: Δh-LIPID-A (kJ/mol)	4.808	-1832.22	W|S	0.9799	1	0.9921	0.8462	0.8939	0.8744
11	2	3	MD: RMSF (A)	P: Δh-LIPID-A (kJ/mol)	2199.87	-1612.36	W|S	0.9798	0.9968	0.9901	0.8519	0.8895	0.8744
12	2	3	D: THR130_B	P: Δh-LIPID-A (kJ/mol)	14.35	-1611.71	W|S	0.9799	1	0.9921	0.849	0.8917	0.8744
13	3	4	P: Δh-SOL (kJ/mol)	D: PHE615_B	0.107	295.042	S|W	0.9847	0.9935	0.9901	0.8519	0.8895	0.8744
14	3	4	P: Δh-IL-GLY (kJ/mol)	D: PHE615_B	0.089	274.717	S|W	0.9847	0.9935	0.9901	0.849	0.8917	0.8744
15	3	4	P: Δh-TAILS (kJ/mol)	D: PHE615_B	0.126	318.541	S|W	0.9848	0.9968	0.9921	0.849	0.8917	0.8744
16	4	8	D: PHE615_B	P: HB-MEM-INTER	-0.03	7.205	W|S	0.9797	0.9935	0.9881	0.8548	0.8874	0.8744
17	1	3	QM: Rotational_constant_c (GHz)	P: Δh-LIPID-A (kJ/mol)	-3704.93	-1013.48	S|W	0.9847	0.9935	0.9901	0.8571	0.8828	0.8727
18	1	3	D: ERR_A20 (kcal/mol)	P: Δh-LIPID-A (kJ/mol)	570.859	-1504.48	W|S	0.9798	0.9968	0.9901	0.8512	0.8871	0.8727
19	1	3	D: ERR_A30 (kcal/mol)	P: Δh-LIPID-A (kJ/mol)	513.93	-1466.44	W|S	0.9798	0.9968	0.9901	0.8512	0.8871	0.8727
20	1	3	D: ERR_B20 (kcal/mol)	P: Δh-LIPID-A (kJ/mol)	328.972	-1376.86	W|S	0.9798	0.9968	0.9901	0.8512	0.8871	0.8727
21	1	3	D: ERR_B30 (kcal/mol)	P: Δh-LIPID-A (kJ/mol)	267.145	-1339.87	W|S	0.9797	0.9935	0.9881	0.8542	0.8849	0.8727
22	1	3	D: #poses_DPB_40%	P: Δh-IL-HEAD	14.536	-2939.74	W|S	0.9798	0.9968	0.9901	0.8427	0.8936	0.8727
23	1	3	D: #poses_DPB_40%	P: HB-WATER-LIPID-A	-0.088	15.326	W|S	0.9798	0.9968	0.9901	0.84	0.8958	0.8727
24	1	3	D: #poses_DPB_40%	P: Δh-LIPID-A (kJ/mol)	8.714	-2226.26	W|S	0.9799	1	0.9921	0.8455	0.8914	0.8727
25	1	12	D: #poses_DPB_40%	QSAR: ASA_P/ASA	-0.003	0.656	W|S	0.9847	0.9935	0.9901	0.8571	0.8828	0.8727
26	1	3	D: THR89_B	P: Δh-LIPID-A (kJ/mol)	4.728	-1383.25	W|S	0.9799	1	0.9921	0.8484	0.8892	0.8727
27	1	3	QSAR: Hararyindex	P: Δh-LIPID-A (kJ/mol)	1.386	-1342.76	W|S	0.9749	0.9967	0.9881	0.8512	0.8871	0.8727
28	2	3	QM: Rotational_constant_a (GHz)	P: Δh-LIPID-A (kJ/mol)	-1019.87	-954.779	S|W	0.9799	1	0.9921	0.8484	0.8892	0.8727
29	3	12	QM: HOMO (Hartree)	QSAR: ASA_P (Å^2)	565.255	402.578	S|W	0.9847	0.9935	0.9901	0.8602	0.8808	0.8727
30	3	4	P: HB-WATER-TAILS	D: PHE615_B	-16.898	238.4	W|S	0.9698	0.9935	0.9842	0.8427	0.8936	0.8727
31	3	4	P: Δh-LIPID-A (kJ/mol)	D: #poses_DPB_30%	0.317	582.916	S|W	0.9799	1	0.9921	0.8484	0.8892	0.8727
32	3	4	P: Δh-LIPID-A (kJ/mol)	D: GLN176_B	0.081	237.763	S|W	0.9799	1	0.9921	0.8455	0.8914	0.8727
33	3	4	P: Δh-LIPID-A (kJ/mol)	D: PHE617_B	0.276	411.109	S|W	0.975	1	0.9901	0.8484	0.8892	0.8727
34	4	12	D: PHE615_B	QSAR: ASA_P/ASA	-0.003	0.781	W|S	0.9747	0.9935	0.9862	0.8571	0.8828	0.8727
35	8	12	P: HB-MEM-INTER	QSAR: ASA_P (Å^2)	-57.579	344.199	W|S	0.9948	0.9841	0.9881	0.8795	0.8688	0.8727
36	1	3	QSAR: Volume (Å^3)	P: Δh-LIPID-A (kJ/mol)	0.607	-1417.18	W|S	0.9747	0.9935	0.9862	0.8506	0.8846	0.8711
37	1	3	MD: Water_1st_hydration_shell	P: Δh-LIPID-A (kJ/mol)	8.039	-1601.42	W|S	0.9798	0.9968	0.9901	0.8477	0.8867	0.8711
38	1	3	MD: ERR_WA1	P: Δh-LIPID-A (kJ/mol)	97.314	-1580.89	W|S	0.9797	0.9935	0.9881	0.8536	0.8825	0.8711
39	1	3	D: Aff_APA_20% (kcal/mol)	P: Δh-LIPID-A (kJ/mol)	-164.919	-2759.12	S|W	0.9749	0.9967	0.9881	0.8449	0.8889	0.8711
40	1	3	D: Aff_APA (kcal/mol)	P: Δh-TAILS (kJ/mol)	-334.734	-4402.66	S|W	0.9796	0.9903	0.9862	0.8477	0.8867	0.8711

Table S7: Top-40 pairs of descriptors and associated ranges with strong (SP) and weak (WP) OM permeation according to the accuracy score of all active compounds. The trained classification model over the compounds in the set G follow the linear equation $y = ax + b$, where y and x are the axes of descriptor i and j, respectively, and they are listed in the measurement units of the descriptors. Column L|R indicates whether WP are associated with descriptor values to the left of the trained linear model and hence SP associated with values to the right of the linear model (entry W|S), or vice versa (entry S|W). The evaluation metrics of positive predictive value (PPV), negative predictive value (NPV) and accuracy (a_0) are listed for each descriptor for the compounds of the set G only and for all active compounds, as indicated.

											set G			all compounds	
r	c_i	c_j	c_k	descriptor i	descriptor j	descriptor k	a	b	c	d	PPV	NPV	a_0	PPV	NPV	a_0
1	3	4	12	P: Δh-CORE-2 (kJ/mol)	D: PHE615_B	QSAR: ASA_H/ASA	-64.547	896.378	-337345.	1574.73	0.99	1.	0.996	0.861	0.9	0.884
2	3	4	12	P: Δh-IL-GLY (kJ/mol)	D: PHE615_B	QSAR: ASA_H/ASA	-56.951	814.238	-316696.	3547.86	1.	0.997	0.998	0.867	0.893	0.883
3	3	4	12	P: Δh-TAILS (kJ/mol)	D: PHE615_B	QSAR: ASA_H/ASA	-74.285	735.046	-309035.	3217.87	0.995	1.	0.998	0.864	0.895	0.883
4	1	3	12	D: Aff_DPB_40% (kcal/mol)	QM: LUMO (Hartree)	QSAR: ASA_H/ASA	-0.13	-2.339	-2.745	0.	0.985	1.	0.994	0.863	0.893	0.881
5	2	4	12	QSAR: Rotatable_bonds	D: PHE615_B	QSAR: ASA_H/ASA	54.446	7.344	-2561.71	-9.696	0.99	0.997	0.994	0.86	0.895	0.881
6	2	4	12	QSAR: Chainbonds	D: PHE615_B	QSAR: ASA_H/ASA	56.138	14.03	-4987.05	24.308	0.99	0.994	0.992	0.863	0.893	0.881
7	3	4	36	P: Δh-LIPID-A (kJ/mol)	D: PHE615_B	QSAR: ASAplus/ASA	-63.269	566.324	-274559.	3152.54	1.	0.994	0.996	0.869	0.889	0.881
8	3	4	12	P: Δh-CORE-1 (kJ/mol)	D: PHE615_B	QSAR: ASA_H/ASA	-42.589	924.569	-299790.	-1005.7	0.995	0.997	0.996	0.86	0.895	0.881
9	1	3	12	D: Aff_DPB_30% (kcal/mol)	QM: LUMO (Hartree)	QSAR: ASA_H/ASA	-0.127	-2.422	-2.712	-0.001	0.98	1.	0.992	0.86	0.893	0.879
10	1	2	12	D: #poses_DPB_40%	QSAR: Rotatable_bonds	QSAR: ASA_H/ASA	6.384	43.123	-1887.3	13.96	0.99	1.	0.996	0.857	0.895	0.879
11	1	3	12	D: #poses_DPB_40%	P: Δh-CORE-2 (kJ/mol)	QSAR: ASA_H/ASA	-312.612	25.767	107021.	1340.04	0.995	1.	0.998	0.86	0.893	0.879
12	1	3	12	D: Aff_DPB (kcal/mol)	QM: LUMO (Hartree)	QSAR: ASA_H/ASA	-0.124	-2.467	-2.664	-0.002	0.975	1.	0.99	0.857	0.895	0.879
13	1	3	36	D: GLN46_B	P: Δh-TAILS (kJ/mol)	QSAR: ASAplus/ASA	228.948	-29.765	-103855.	-6.769	0.995	0.994	0.994	0.86	0.893	0.879
14	1	4	12	QSAR: Plattindex	D: PHE178_B	QSAR: ASA_H/ASA	43.512	48.02	-27192.8	-102.343	0.995	0.997	0.996	0.866	0.888	0.879
15	1	4	12	QSAR: Surface (Å^2)	D: PHE615_B	QSAR: ASA_H/ASA	34.629	278.931	-101122.	-191.046	0.985	0.997	0.992	0.857	0.895	0.879
16	2	4	12	MD: ERR_MPA (Å^2)	D: PHE615_B	QSAR: ASA_H/ASA	33.809	4.79	-1476.4	-3.831	0.99	0.994	0.992	0.86	0.893	0.879
17	2	4	12	MD: ERR_ACY	D: PHE615_B	QSAR: ASA_H/ASA	43.931	0.255	-94.278	0.712	0.995	0.987	0.99	0.869	0.886	0.879
18	2	4	12	P: Δs-LIPID-A (J/mol K)	D: PHE615_B	QSAR: ASA_H/ASA	37.815	355.474	-115024.	-1188.95	0.985	0.997	0.992	0.857	0.895	0.879
19	3	4	12	QM: HOMO (Hartree)	D: PHE615_B	QSAR: ASA_H/ASA	-52.496	0.134	-59.11	0.128	0.995	0.997	0.996	0.866	0.888	0.879
20	3	4	12	QM: LUMO (Hartree)	D: PHE615_B	QSAR: ASA_H/ASA	-53.461	0.184	-59.362	-0.872	0.99	1.	0.996	0.854	0.897	0.879
21	3	4	12	P: Δh-SOL (kJ/mol)	D: PHE615_B	QSAR: ASA_H/ASA	-63.562	731.009	-298440.	5726.87	0.995	0.994	0.994	0.863	0.89	0.879
22	3	4	12	P: Δh-IL-HEAD	D: PHE615_B	QSAR: ASA_H/ASA	-66.683	685.875	-306706.	6974.82	0.995	0.997	0.996	0.863	0.89	0.879
23	3	4	12	P: Δh-LIPID-A (kJ/mol)	D: PHE615_B	QSAR: ASA_H/ASA	-78.304	668.34	-302908.	2764.13	0.995	1.	0.998	0.863	0.89	0.879
24	3	4	12	P: Δh-CORE-1 (kJ/mol)	D: #poses_DPB_30%	QSAR: ASA_H/ASA	-73.633	724.374	-379461.	6888.94	0.985	0.994	0.99	0.863	0.89	0.879
25	1	3	12	D: Aff_DPB_20% (kcal/mol)	QM: LUMO (Hartree)	QSAR: ASA_H/ASA	-0.123	-2.428	-2.695	0.004	0.995	0.99	0.992	0.868	0.884	0.878
26	1	3	12	D: #poses_DPB_40%	P: Δh-IL-HEAD	QSAR: ASA_H/ASA	-698.75	30.461	181346.	-420.221	0.99	1.	0.996	0.853	0.894	0.878
27	1	3	12	D: #poses_DPB_40%	P: Δh-IL-GLY (kJ/mol)	QSAR: ASA_H/ASA	-350.939	27.876	111184.	2597.91	0.995	1.	0.998	0.859	0.89	0.878
28	1	3	12	D: #poses_DPB_40%	P: Δh-LIPID-A (kJ/mol)	QSAR: ASA_H/ASA	-309.314	24.115	98180.8	164.15	0.995	1.	0.998	0.859	0.89	0.878
29	1	3	36	D: #poses_DPB_40%	P: Δh-CORE-2 (kJ/mol)	QSAR: ASAplus/ASA	260.738	-19.013	-88564.3	365.962	0.995	0.997	0.996	0.856	0.892	0.878
30	1	4	12	D: GLN46_B	D: PHE615_B	QSAR: ASA_H/ASA	56.089	102.781	-37337.1	118.866	0.995	0.99	0.992	0.865	0.886	0.878
31	1	3	12	D: ARG620_B	QM: HOMO (Hartree)	QSAR: ASA_H/ASA	-0.123	36.397	40.731	-0.524	0.99	0.997	0.994	0.853	0.894	0.878
32	1	4	12	QSAR: Randicindex	D: PHE178_B	QSAR: ASA_H/ASA	51.036	4.448	-3388.78	19.364	0.995	0.99	0.992	0.868	0.884	0.878
33	1	4	12	QM: E_thermal (kcal/mol)	D: #poses_DPB_30%	QSAR: ASA_H/ASA	56.572	88.556	-58795.5	-30.918	0.995	0.997	0.996	0.856	0.892	0.878
34	2	4	12	MD: MPA (Å^2)	D: PHE615_B	QSAR: ASA_H/ASA	46.023	29.777	-12126.4	49.277	0.995	0.99	0.992	0.868	0.884	0.878
35	2	4	12	MD: ERR_MPA (Å^2)	D: GLN176_B	QSAR: ASA_H/ASA	26.861	4.741	-1229.01	-6.514	0.975	0.994	0.986	0.853	0.894	0.878
36	2	4	12	MD: ERR_ACY	D: GLN176_B	QSAR: ASA_H/ASA	43.017	0.226	-75.808	-0.277	0.98	0.997	0.99	0.853	0.894	0.878
37	2	4	12	QSAR: Chainatoms	D: PHE615_B	QSAR: ASA_H/ASA	62.152	12.995	-4981.36	54.159	0.995	0.987	0.99	0.868	0.884	0.878
38	2	4	12	P: Δs-IL-GLY (J/mol K)	D: PHE615_B	QSAR: ASA_H/ASA	37.517	431.642	-141139.	-977.553	0.99	0.99	0.99	0.859	0.89	0.878
39	3	8	12	QM: HOMO (Hartree)	P: HB-MEM-INTER	QSAR: ASA_H/ASA	-0.638	0.116	-0.606	-0.018	0.985	0.994	0.99	0.865	0.886	0.878
40	3	4	36	P: Δh-TAILS (kJ/mol)	D: PHE136_B	QSAR: ASAplus/ASA	-60.59	241.588	-155730.	1704.63	0.99	0.994	0.992	0.859	0.89	0.878

Table S8: Top-40 groups of three descriptors and associated ranges with strong (SP) and weak (WP) OM permeation according to the accuracy score of all active compounds. The trained classification model over the compounds in the set G follow the equation $ax + by + cz + d = 0$, where x, y, and z are the axes of descriptors i, j, and k, respectively, and they are listed in the measurement units of the descriptors. The evaluation metrics of positive predictive value (PPV), negative predictive value (NPV) and accuracy (a_0) are listed for each descriptor for the compounds of the set G only and for all active compounds, as indicated.

Supplementary References

[1] J. Mehla, G. Malloci, R. Mansbach, C.A. López, R. Tsivkovski, K. Haynes, I.V. Leus, S.B. Grindstaff, R.H. Cascella, N. D'Cunha, L. Herndon, N.W. Hengartner, E. Margiotta, A. Atzori, A.V. Vargiu, P.D. Manrique, J.K. Walker, O. Lomovskaya, P. Ruggerone, S. Gnanakaran, V.V. Rybenkov, H.I. Zgurskaya. Predictive Rules of Efflux Inhibition and Avoidance in *Pseudomonas aeruginosa. mBio.* **12** (1): e02785-20, (2021)

[2] Rachael A. Mansbach, Inga V. Leus, Jitender Mehla, Cesar A. Lopez, John K. Walker, Valentin V. Rybenkov, Nicolas W. Hengartner, Helen I. Zgurskaya, and S. Gnanakaran. Machine Learning Algorithm Identifies an Antibiotic Vocabulary for Permeating Gram-Negative Bacteria. *Journal of Chemical Information and Modeling.* **60**, 6, 2838-2847, (2020)

[3] ChemAxon. 2017. Marvin suite. https://chemaxon.com.

[4] Gaussian 16, Revision C.01, M. J. Frisch, G. W. Trucks, H. B. Schlegel, G. E. Scuseria, M. A. Robb, J. R. Cheeseman, G. Scalmani, V. Barone, G. A. Petersson, H. Nakatsuji, X. Li, M. Caricato, A. V. Marenich, J. Bloino, B. G. Janesko, R. Gomperts, B. Mennucci, H. P. Hratchian, J. V. Ortiz, A. F. Izmaylov, J. L. Sonnenberg, D. Williams-Young, F. Ding, F. Lipparini, F. Egidi, J. Goings, B. Peng, A. Petrone, T. Henderson, D. Ranasinghe, V. G. Zakrzewski, J. Gao, N. Rega, G. Zheng, W. Liang, M. Hada, M. Ehara, K. Toyota, R. Fukuda, J. Hasegawa, M. Ishida, T. Nakajima, Y. Honda, O. Kitao, H. Nakai, T. Vreven, K. Throssell, J. A. Montgomery, Jr., J. E. Peralta, F. Ogliaro, M. J. Bearpark, J. J. Heyd, E. N. Brothers, K. N. Kudin, V. N. Staroverov, T. A. Keith, R. Kobayashi, J. Normand, K. Raghavachari, A. P. Rendell, J. C. Burant, S. S. Iyengar, J. Tomasi, M. Cossi, J. M. Millam, M. Klene, C. Adamo, R. Cammi, J. W. Ochterski, R. L. Martin, K. Morokuma, O. Farkas, J. B. Foresman, and D. J. Fox, Gaussian, Inc., Wallingford CT, 2016.

[5] Malloci, G.; Vargiu, A.V.; Serra, G.; Bosin, A.; Ruggerone, P.; Ceccarelli, M. A Database of Force-Field Parameters, Dynamics, and Properties of Antimicrobial Compounds. *Molecules.* **20**, 13997-14021 (2015).

[6] Singh, U.C. and Kollman, P.A. An approach to computing electrostatic charges for molecules. *J. Comput. Chem.*, **5** 129-145 (1984)

[7] Christopher I. Bayly, Piotr Cieplak, Wendy Cornell, and Peter A. Kollman. *The Journal of Physical Chemistry*, **97**, 40, 10269-10280 (1993)

[8] J. Wang, W. Wang, P.A. Kollman, D.A. Case. Automatic atom type and bond type perception in molecular mechanical calculations. *Journal of Molecular Graphics and Modelling*, **25**, 2, 247-260, (2006)

[9] Wang, J., Wolf, R.M., Caldwell, J.W., Kollman, P.A. and Case, D.A., Development and testing of a general amber force field. *J. Comput. Chem.*, **25**, 1157-1174, (2004)

[10] D.R. Roe and T.E. Cheatham. *Journal of Chemical Theory and Computation*, **9**, 7, 3084-3095 (2013)

[11] J. Shao, S.W. Tanner, N. Thompson, and Thomas E. Cheatham. *Journal of Chemical Theory and Computation.* **3**, 6, 2312-2334 (2007)

[12] Trott, O. and Olson, A.J. AutoDock Vina: Improving the speed and accuracy of docking with a new scoring function, efficient optimization, and multithreading. *J. Comput. Chem.*, **31**, 455-461 (2010)

[13] Morris, G.M., Huey, R., Lindstrom, W., Sanner, M.F., Belew, R.K., Goodsell, D.S. and Olson, A.J. (2009), AutoDock4 and AutoDockTools4: Automated docking with selective receptor flexibility. *J. Comput. Chem.*, **30**, 2785-2791 (2009)

[14] G. Sennhauser, M.A. Bukowska, C. Briand, M.G. Grütter. Crystal Structure of the Multidrug Exporter MexB from *Pseudomonas aeruginosa*, *Journal of Molecular Biology*, **389**, 1, 134-145, (2009)

[15] Nakashima, R., Sakurai, K., Yamasaki, S. et al. Structural basis for the inhibition of bacterial multidrug exporters. *Nature* **500**, 102–106 (2013)

[16] Ramaswamy Venkata K., Vargiu Attilio V., Malloci Giuliano, Dreier Jürg, Ruggerone Paolo. Molecular Determinants of the Promiscuity of MexB and MexY Multidrug Transporters of *Pseudomonas aeruginosa*, *Frontiers in Microbiology*, **9**, (2018)

[17] Alessio Atzori, Viveka N. Malviya, Giuliano Malloci, Jürg Dreier, Klaas M. Pos, Attilio V. Vargiu, Paolo Ruggerone. Identification and characterization of carbapenem binding sites within the RND-transporter AcrB. *Biochimica et Biophysica Acta (BBA) - Biomembranes*,**1861**, 1, 62-74, (2019)

[18] Kirschner, K. N. et al. GLYCAM06: a generalizable biomolecular force field. Carbohydrates. J Comput Chem 29, 622–655 (2008).

[19] P'all, S., Abraham, M. J., Kutzner, C., Hess, B., Lindahl, E. in Solving Software Challenges for Exascale (eds. Markidis, S., Laure, E.) 8759, 3–27 (Springer International Publishing, 2014).

[20] Chen, F. and Smith, P. E. Simulated surface tensions of common water models. J. Chem. Phys. 126, 221101 (2007).

[21] Kirschner, K. N., Lins, R. D., Maass, A. A glycam-based force field for simulations of lipopolysaccharide membranes: parametrization and validation. J. Chem. Theory and Comput. (2012). doi:10.1021/ct300534j

[22] Tomasi J., Mennucci B., Cammi R. Quantum Mechanical Continuum Solvation Models. *Chemical Reviews* **105** 8, 2999-3094 (2005)

[23] Singh, U.C. and Kollman, P.A., An approach to computing electrostatic charges for molecules. *J. Comput. Chem.*, **5**, 129-145 (1984)

[24] Bayly, C.I., Cieplak P., Cornell W., Kollman P.A. A well-behaved electrostatic potential based method using charge restraints for deriving atomic charges: the RESP model. *The Journal of Physical Chemistry.* **97** 40, 10269-10280 (1993)

[25] Wang J., Wang W., Kollman P.A., Case D.A.. Automatic atom type and bond type perception in molecular mechanical calculations. *J. Mol. Graph. Model.* **25**, 247-260, (2006)

[26] Wang, J., Wolf, R. M., Caldwell, J. W., Kollman, P. A., Case, D. A. Development and testing of a general amber force field. J Comput Chem 25, 1157–1174 (2004).

[27] Sousa da Silva, A. W., Vranken, W. F. ACPYPE - AnteChamber PYthon Parser interfacE. BMC Res Notes 5, 367 (2012).

[28] Hess, B. LINCS: A Parallel Linear Constraint Solver for Molecular Simulation. J Chem Theory Comput 4, 116–122 (2008).

[29] Tironi, I. G., Sperb, R., Smith, P. E. A generalized reaction field method for molecular dynamics simulations. The Journal of chemical … (1995).

[30] Berendsen, H. J. C., Postma, J. P. M., van Gunsteren, W. F., DiNola, A., Haak, J. R. Molecular dynamics with coupling to an external bath. J. Chem. Phys. 81, 3684–3684 (1984).

[31] Parrinello, M., RAHMAN, A. Polymorphic transitions in single crystals: A new molecular dynamics method. Journal of Applied Physics 52, 7182–7190 (1981).

[32] Bussi, G., Donadio, D., Parrinello, M. Canonical sampling through velocity rescaling. J. Chem. Phys. 126, 014101 (2007).

[33] Strateva, T. Yordanov, D. Pseudomonas aeruginosa - a phenomenon of bacterial resistance. *J Med Microbiol* **58**, 1133-1148, (2009)

[34] Chatterjee, M. *et al.* Antibiotic resistance in Pseudomonas aeruginosa and alternative therapeutic options. *Int J Med Microbiol* **306**, 48-58, (2016)

[35] Breidenstein, E.B., de la Fuente-Nunez, C., Hancock, R.E. Pseudomonas aeruginosa: all roads lead to resistance. *Trends Microbiol* **19**, 419-426, (2011)

[36] Ilyas Alav, Jessica Kobylka, Miriam S. Kuth, Klaas M. Pos, Martin Picard, Jessica M. A. Blair, and Vassiliy N. Bavro. Structure, Assembly, and Function of Tripartite Efflux and Type 1 Secretion Systems in Gram-Negative Bacteria. *Chemical Reviews* **121** (9), 5479-5596, (2021)

[37] P.A. Klenotic, M.A. Moseng, C.E. Morgan, and E.W.Yu. Structural and Functional Diversity of Resistance–Nodulation–Cell Division Transporters. *Chemical Reviews* **121** (9), 5378-5416 (2021)

[38] W. S. Noble. What is support vector machine? *Nature Biotechnology*, **24**, 12, (2006)
