# Supplementary material for: Predicting permeation of compounds across the outer membrane of P. aeruginosa using molecular descriptors
Source: Commun Chem. 2024 Apr 12;7:84. doi: 10.1038/s42004-024-01161-y (PMC11015012; doi:10.1038/s42004-024-01161-y)
Supplement: Supplementary file 2 — Description of Additional Supplementary Files [file 42004_2024_1161_MOESM2_ESM.pdf]

Description of Additional Supplementary Files

File name: Supplementary Data 1

Description: library of compounds used in this study, including chemotype, SMILES, descriptor values and IC50's

File name: Supplementary Data 2

Description: definitions of all molecular descriptors calculated in this study
