# Supplementary material for: Predicting permeation of compounds across the outer membrane of P. aeruginosa using molecular descriptors
Source: Commun Chem. 2024 Apr 12;7:84. doi: 10.1038/s42004-024-01161-y (PMC11015012; doi:10.1038/s42004-024-01161-y)
Supplement: Supplementary file 4 — Supplementary Data 2 [file 42004_2024_1161_MOESM4_ESM.docx]

**Supplementary Data 2. Descriptor Definitions.**

| Name | Unit | Description |
| --- | --- | --- |
| Molecular_weight | (g/mol) | Molecular weight of the molecule as computed with cxcalc (https://chemaxon.com/marvin-archive/latest/help/applications/cxcalc-calculations.html). |
| Total_charge | (e) | Total molecular charge of the micro-species considered. |
| Atom_Count |  | Total number of atoms as computed with cxcalc (https://chemaxon.com/marvin-archive/latest/help/applications/cxcalc-calculations.html). |
| Heavy_atoms |  | Total number of non-Hydrogen atoms as computed with cxcalc (https://chemaxon.com/marvin-archive/latest/help/applications/cxcalc-calculations.html). |
| Asymmetric_atoms |  | Asymmetric atoms as computed with cxcalc (https://chemaxon.com/marvin-archive/latest/help/applications/cxcalc-calculations.html). |
| Total_DFT_energy | (Hartree) | Total energy from density functional theory (DFT, B3LYP-6-31G**) single-point calculation in vacuum (at the molecular configuration optimized in implicit solvent). |
| HL_gap | (Hartree) | HOMO-LUMO energy gap: difference between the energies associated to the frontier molecular orbitals HOMO and LUMO. |
| Total_dipole_moment | (Db) | Total dipole moment of the molecule extracted from single-point energy calculation in vacuum (at the DFT optimized geometry in implicit solvent). This value is consistent with the atomic partial charges adopted for molecular dynamics simulations in explicit water solvent. |
| Rotational_constant_a | (GHz) | Rotational constant A computed at the molecular configuration optimized in implicit solvent. |
| Rotational_constant_b | (GHz) | Rotational constant B computed at the molecular configuration optimized in implicit solvent. |
| Rotational_constant_c | (GHz) | Rotational constant C computed at the molecular configuration optimized in implicit solvent. |
| Volume | (Å^3^) | van der Waals volume computed at the DFT optimized geometry in implicit solvent. |
| Water_1st_hydration_shell |  | Average number of water molecules in the first solvation shell as extracted from micro-second long molecular dynamics simulations in explicit water solvent. |
| ERR_WA1 |  | Standard deviation associated to Water_1st_hydration_shell. |
| Water_2nd_hydration_shell |  | Average number of water molecules in the second solvation shell as extracted from micro-second long molecular dynamics simulations in explicit water solvent. |
| ERR_WA2 |  | Standard deviation associated to Water_2nd_hydration_shell. |
| RMSF | (Å) | Average value of the root mean square fluctuation of atomic positions in the molecular dynamics trajectory. |
| ERR_RMSF | (Å) | Standard deviation associated to RMSF. |
| MPA | (Å^2^) | Average value of the minimal projection area associated to the configurations explored by the molecule during MD trajectory. |
| ERR_MPA | (Å^2^) | Standard deviation associated to MPA. |
| Asphericity |  | Average asphericity computed along MD trajectory. This parameter gives a measure of the deviation of the mass distribution from spherical symmetry. |
| ERR_ASP |  | Standard deviation associated to asphericity. |
| Acylindricity |  | Average acylindricity computed along MD trajectory. This parameter gives a measure of the deviation of the mass distribution from cylindrical symmetry. |
| ERR_ACY |  | Standard deviation associated to acylindricity. |
| Kappa2 |  | Average relative shape anisotropy kappa2 computed along MD trajectory. This parameter is limited between 0 and 1 and reflects both symmetry and dimensionality of the molecule. |
| ERR_K2 |  | Standard deviation associated to kappa2. |
| #poses_APA_20% |  | Total number of docking poses inside the access pocket of the access monomer of MexB (APA) in contact with at least 20% of the residues lining the pocket. |
| Aff_APA_20% | (kcal/mol) | Average value of the free energy of binding extracted from molecular docking for the #poses_APA_20% poses. |
| ERR_A20 | (kcal/mol) | Standard deviation associated to Aff_APA_20%. |
| #poses_APA_30% |  | Total number of docking poses inside the APA in contact with at least 30% of the residues lining the pocket. |
| Aff_APA_30% | (kcal/mol) | Average value of the free energy of binding extracted from molecular docking for the #poses_APA_30% poses. |
| ERR_A30 | (kcal/mol) | Standard deviation associated to Aff_APA_30%. |
| #poses_APA_40% |  | Total number of docking poses inside the APA in contact with at least 40% of the residues lining the pocket. |
| Aff_APA_40% | (kcal/mol) | Average value of the free energy of binding extracted from molecular docking for the #poses_APA_40% poses. |
| ERR_A40 | (kcal/mol) | Standard deviation associated to Aff_APA_40%. |
| Aff_APA | (kcal/mol) | Average value of the free energy of binding extracted from molecular docking for all docking poses found in the APA. |
| ERR_APA | (kcal/mol) | Standard deviation associated to Aff_APA. |
| SER79_A |  | Total number of contacts to residue SER79 of the APA considering only #poses_APA_30%. |
| THR91_A |  | Total number of contacts to residue THR91 of the APA considering only #poses_APA_30%. |
| LYS134_A |  | Total number of contacts to residue LYS134 of the APA considering only #poses_APA_30%. |
| ASN135_A |  | Total number of contacts to residue ASN135 of the APA considering only #poses_APA_30%. |
| LYS292_A |  | Total number of contacts to residue LYS292 of the APA considering only #poses_APA_30%. |
| PHE573_A |  | Total number of contacts to residue PHE573 of the APA considering only #poses_APA_30%. |
| GLN575_A |  | Total number of contacts to residue GLN575 of the APA considering only #poses_APA_30%. |
| GLN577_A |  | Total number of contacts to residue GLN577 of the APA considering only #poses_APA_30%. |
| PHE617_A |  | Total number of contacts to residue PHE617 of the APA considering only #poses_APA_30%. |
| SER624_A |  | Total number of contacts to residue SER624 of APA considering only #poses_APA_30%. |
| MET662_A |  | Total number of contacts to residue MET662 of the APA considering only #poses_APA_30%. |
| PHE664_A |  | Total number of contacts to residue PHE664 of the APA considering only #poses_APA_30%. |
| PHE666_A |  | Total number of contacts to residue PHE666 of the APA considering only #poses_APA_30%. |
| ALA667_A |  | Total number of contacts to residue ALA667 of the APA considering only #poses_APA_30%. |
| PRO668_A |  | Total number of contacts to residue PRO668 of the APA considering only #poses_APA_30%. |
| LEU674_A |  | Total number of contacts to residue LEU674 of the APA considering only #poses_APA_30%. |
| ASN676_A |  | Total number of contacts to residue ASN676 of the APA considering only #poses_APA_30%. |
| ASP681_A |  | Total number of contacts to residue ASP681 of the APA considering only #poses_APA_30%. |
| ARG716_A |  | Total number of contacts to residue ARG716 of the APA considering only #poses_APA_30%. |
| ASN718_A |  | Total number of contacts to residue ASN718 of the APA considering only #poses_APA_30%. |
| GLU825_A |  | Total number of contacts to residue GLU825 of the APA considering only #poses_APA_30%. |
| #poses_DPB_20% |  | Total number of docking poses inside the deep pocket of the binding monomer of MexB (DPB) in contact with at least 20% of the residues lining the pocket. |
| Aff_DPB_20% | (kcal/mol) | Average value of the free energy of binding extracted from molecular docking for the #poses_DPB_20% poses. |
| ERR_B20 | (kcal/mol) | Standard deviation associated to Aff_DPB_20%. |
| #poses_DPB_30% |  | Total number of docking poses inside the DPB in contact with at least 30% of the residues lining the pocket. |
| Aff_DPB_30% | (kcal/mol) | Average value of the free energy of binding extracted from molecular docking for the #poses_DPB_30% poses. |
| ERR_B30 | (kcal/mol) | Standard deviation associated to Aff_DPB_30%. |
| #poses_DPB_40% |  | Total number of docking poses inside the DPB in contact with at least 40% of the residues lining the pocket. |
| Aff_DPB_40% | (kcal/mol) | Average value of the free energy of binding extracted from molecular docking for the #poses_DPB_40% poses. |
| ERR_B40 | (kcal/mol) | Standard deviation associated to Aff_DPB_40%. |
| Aff_DPB | (kcal/mol) | Average value of the free energy of binding extracted from molecular docking for all docking poses found in the DPB. |
| ERR_DPB | (kcal/mol) | Standard deviation associated to Aff_DPB. |
| GLN46_B |  | Total number of contacts to residue GLN46 of the DPB considering only #poses_DPB_30%. |
| THR89_B |  | Total number of contacts to residue THR89 of the DPB considering only #poses_DPB_30%. |
| ARG128_B |  | Total number of contacts to residue ARG128 of the DPB considering only #poses_DPB_30%. |
| THR130_B |  | Total number of contacts to residue THR130 of the DPB considering only #poses_DPB_30%. |
| LYS134_B |  | Total number of contacts to residue LYS134 of the DPB considering only #poses_DPB_30%. |
| PHE136_B |  | Total number of contacts to residue PHE136 of the DPB considering only #poses_DPB_30%. |
| VAL139_B |  | Total number of contacts to residue VAL139 of the DPB considering only #poses_DPB_30%. |
| GLN176_B |  | Total number of contacts to residue GLN176 of the DPB considering only #poses_DPB_30%. |
| VAL177_B |  | Total number of contacts to residue VAL177 of the DPB considering only #poses_DPB_30%. |
| PHE178_B |  | Total number of contacts to residue PHE178 of the DPB considering only #poses_DPB_30%. |
| GLY179_B |  | Total number of contacts to residue GLY179 of the DPB considering only #poses_DPB_30%. |
| SER180_B |  | Total number of contacts to residue SER180 of the DPB considering only #poses_DPB_30%. |
| GLN273_B |  | Total number of contacts to residue GLN273 of the DPB considering only #poses_DPB_30%. |
| ASP274_B |  | Total number of contacts to residue ASP274 of the DPB considering only #poses_DPB_30%. |
| SER276_B |  | Total number of contacts to residue SER276 of the DPB considering only #poses_DPB_30%. |
| ILE277_B |  | Total number of contacts to residue ILE277 of the DPB considering only #poses_DPB_30%. |
| TYR327_B |  | Total number of contacts to residue TYR327 of the DPB considering only #poses_DPB_30%. |
| PHE573_B |  | Total number of contacts to residue PHE573 of the DPB considering only #poses_DPB_30%. |
| PHE610_B |  | Total number of contacts to residue PHE610 of the DPB considering only #poses_DPB_30%. |
| VAL612_B |  | Total number of contacts to residue VAL612 of the DPB considering only #poses_DPB_30%. |
| PHE615_B |  | Total number of contacts to residue PHE615 of the DPB considering only #poses_DPB_30%. |
| PHE617_B |  | Total number of contacts to residue PHE617 of the DPB considering only #poses_DPB_30%. |
| ARG620_B |  | Total number of contacts to residue ARG620 of the DPB considering only #poses_DPB_30%. |
| PHE628_B |  | Total number of contacts to residue PHE628 of the DPB considering only #poses_DPB_30%. |
| Rotatable_bonds |  | Rotatable bonds as computed with cxcalc (https://chemaxon.com/marvin-archive/latest/help/applications/cxcalc-calculations.html). |
| Aliphatic_atoms |  | Aliphatic atoms as computed with cxcalc (https://chemaxon.com/marvin-archive/latest/help/applications/cxcalc-calculations.html). |
| Aromatic_atoms |  | Aromatic atoms as computed with cxcalc (https://chemaxon.com/marvin-archive/latest/help/applications/cxcalc-calculations.html). |
| Bonds |  | Bonds as computed with cxcalc (https://chemaxon.com/marvin-archive/latest/help/applications/cxcalc-calculations.html). |
| Aliphatic_bonds |  | Aliphatic bonds as computed with cxcalc (https://chemaxon.com/marvin-archive/latest/help/applications/cxcalc-calculations.html). |
| Aromatic_bonds |  | Aromatic bonds as computed with cxcalc (https://chemaxon.com/marvin-archive/latest/help/applications/cxcalc-calculations.html). |
| Rings |  | Rings as computed with cxcalc (https://chemaxon.com/marvin-archive/latest/help/applications/cxcalc-calculations.html). |
| Aliphatic_rings |  | Aliphatic rings as computed with cxcalc (https://chemaxon.com/marvin-archive/latest/help/applications/cxcalc-calculations.html). |
| Aromatic_rings |  | Aromatic rings as computed with cxcalc (https://chemaxon.com/marvin-archive/latest/help/applications/cxcalc-calculations.html). |
| Heterorings |  | Heterorings as computed with cxcalc (https://chemaxon.com/marvin-archive/latest/help/applications/cxcalc-calculations.html). |
| Chainatoms |  | Chain atoms as computed with cxcalc (https://chemaxon.com/marvin-archive/latest/help/applications/cxcalc-calculations.html). |
| Chainbonds |  | Chainbonds as computed with cxcalc (https://chemaxon.com/marvin-archive/latest/help/applications/cxcalc-calculations.html). |
| Refractivity |  | Refractivity as computed with cxcalc (https://chemaxon.com/marvin-archive/latest/help/applications/cxcalc-calculations.html). |
| Balabanindex |  | Balabanindex as computed with cxcalc (https://chemaxon.com/marvin-archive/latest/help/applications/cxcalc-calculations.html). |
| Hararyindex |  | Hararyindex as computed with cxcalc (https://chemaxon.com/marvin-archive/latest/help/applications/cxcalc-calculations.html). |
| Plattindex |  | Plattindex as computed with cxcalc (https://chemaxon.com/marvin-archive/latest/help/applications/cxcalc-calculations.html). |
| Randicindex |  | Randicindex as computed with cxcalc (https://chemaxon.com/marvin-archive/latest/help/applications/cxcalc-calculations.html). |
| Szegedindex |  | Szegedindex as computed with cxcalc (https://chemaxon.com/marvin-archive/latest/help/applications/cxcalc-calculations.html). |
| Wienerindex |  | Wienerindex as computed with cxcalc (https://chemaxon.com/marvin-archive/latest/help/applications/cxcalc-calculations.html). |
| Hyperwienerindex |  | Hyperwienerindex as computed with cxcalc (https://chemaxon.com/marvin-archive/latest/help/applications/cxcalc-calculations.html). |
| Donors |  | Donors as computed with cxcalc (https://chemaxon.com/marvin-archive/latest/help/applications/cxcalc-calculations.html). |
| Acceptors |  | Acceptors as computed with cxcalc (https://chemaxon.com/marvin-archive/latest/help/applications/cxcalc-calculations.html). |
| Resonantcount |  | Resonantcount as computed with cxcalc (https://chemaxon.com/marvin-archive/latest/help/applications/cxcalc-calculations.html). |
| Surface | (Å^2^) | Molecular surface area (3D) as computed with cxcalc (https://chemaxon.com/marvin-archive/latest/help/applications/cxcalc-calculations.html). |
| Topological_surface | (Å^2^) | Topological surface area (2D) as computed with cxcalc (https://chemaxon.com/marvin-archive/latest/help/applications/cxcalc-calculations.html). |
| ASA | (Å^2^) | Water accessible surface area as computed with cxcalc (https://chemaxon.com/marvin-archive/latest/help/applications/cxcalc-calculations.html). |
| ASAplus | (Å^2^) | Positive accessible surface area as computed with cxcalc (https://chemaxon.com/marvin-archive/latest/help/applications/cxcalc-calculations.html). |
| ASAminus | (Å^2^) | Negative accessible surface area as computed with cxcalc (https://chemaxon.com/marvin-archive/latest/help/applications/cxcalc-calculations.html). |
| ASA_H | (Å^2^) | Total hydrophobic surface area as computed with cxcalc (https://chemaxon.com/marvin-archive/latest/help/applications/cxcalc-calculations.html). |
| ASA_P | (Å^2^) | Total polar surface area as computed with cxcalc (https://chemaxon.com/marvin-archive/latest/help/applications/cxcalc-calculations.html). |
| ASAplus/ASA |  | Ratio ASAplus/ASA computed with cxcalc (https://chemaxon.com/marvin-archive/latest/help/applications/cxcalc-calculations.html). |
| ASAminus/ASA |  | Ratio ASAminus/ASA computed with cxcalc (https://chemaxon.com/marvin-archive/latest/help/applications/cxcalc-calculations.html). |
| ASA_H/ASA |  | Ratio ASA_H/ASA computed with cxcalc (https://chemaxon.com/marvin-archive/latest/help/applications/cxcalc-calculations.html). |
| ASA_P/ASA |  | Ratio ASA_P/ASA computed with cxcalc (https://chemaxon.com/marvin-archive/latest/help/applications/cxcalc-calculations.html). |
| Pienergy |  | Pi energy of the molecule as computed with cxcalc (https://chemaxon.com/marvin-archive/latest/help/applications/cxcalc-calculations.html). |
| FSP3 |  | Fraction of sp3 carbons of the molecule as computed with cxcalc (https://chemaxon.com/marvin-archive/latest/help/applications/cxcalc-calculations.html). |
| Cyclomatic_number |  | Cyclomatic number as computed with cxcalc (https://chemaxon.com/marvin-archive/latest/help/applications/cxcalc-calculations.html). |
| xlogP3 |  | Octanol-water partition coefficient as computed with the xlogp3 software (http://www.sioc-ccbg.ac.cn/?p=42&software=xlogp3). |
| logP |  | logP as computed with cxcalc (https://chemaxon.com/marvin-archive/latest/help/applications/cxcalc-calculations.html) |
| logD |  | logD at pH=7.4 as computed with cxcalc (https://chemaxon.com/marvin-archive/latest/help/applications/cxcalc-calculations.html) |
| HOMO | (Hartree) | Energy associated to the highest occupied molecular orbital (HOMO) in the DFT calculation in vacuum at the molecular configuration optimized in implicit solvent. |
| LUMO | (Hartree) | Energy associated to the lowest unoccupied molecular orbital (LUMO) in the DFT calculation in vacuum at the molecular configuration optimized in implicit solvent. |
| Isotropic_pol | (a.u.) | Isotropic average of dipole polarizability from polarizability tensor computed with Gaussian. |
| Anisotropic_pol | (a.u.) | Anisotropic average of dipole polarizability from polarizability tensor computed with Gaussian. |
| E_thermal | (kcal/mol) | Internal thermal energy from thermochemical analysis in Gaussian. |
| CV | (cal/mol-Kelvin) | Constant-volume heat capacity from thermochemical analysis in Gaussian. |
| S | (cal/mol-Kelvin) | Entropy from thermochemical analysis in Gaussian. |
| HB-WATER |  | Total number of hydrogen bonds with water molecules. |
| HB-MEM-INTER |  | Total number of hydrogen bonds with regions of the outer membrane (water-membrane interface inner leaflet). |
| Δxy-SOL (cm2/s) |  | Lateral diffusion constant, assuming membrane is placed in the XY plane. |
| Δh-SOL (kJ/mol) | (kJ/mol) | Total interaction energy (Coulomb, LJ) with the water molecules. |
| Δs-SOL (J/mol K) | (J/mol K) | Cumulative entropy within the water interface. |
| HB-IL-HEAD |  | Total number of hydrogen bonds with the head groups (choline, phosphate) of the outer membrane inner leaflet. |
| HB-WATER-IL-HEAD |  | Total number of hydrogen bonds with water molecules in the head group region of the outer membrane inner leaflet. |
| Δxy-IL-HEAD (cm2/s) |  | Lateral diffusion coefficient within the head groups of the outer membrane inner leaflet. |
| Δh-IL-HEAD |  | Total energy (Coulomb, LJ) with the head groups of the outer membrane inner leaflet. |
| Δs-IL-HEAD (J/mol K) | (J/mol K) | Cumulative entropy of the molecule in the head group region of the outer membrane inner leaflet. |
| HB-IL-GLY |  | Total number of hydrogen bonds within the glycerol moiety region of the outer membrane inner leaflet. |
| HB-WATER-IL-GLY |  | Total number of hydrogen bonds with eater molecules in the glycerol moiety of the outer membrane inner leaflet. |
| Δxy-IL-GLY (cm2/s) |  | Lateral diffusion coefficient in the glycerol moiety region of the outer membrane inner leaflet. |
| Δh-IL-GLY (kJ/mol) |  | Total interaction energy (Coulomb, LJ) with the glycerol moiety region of the outer membrane inner leaflet. |
| Δs-IL-GLY (J/mol K) | (J/mol K) | Cumulative entropy of the drug within the glycerol moiety region of the outer membrane inner leaflet. |
| HB-TAILS |  | Number of hydrogen bonds with the membrane within the aliphatic tail region of the outer membrane. |
| HB-WATER-TAILS |  | Total number of hydrogen bonds with water molecules (if present) within the aliphatic tails of the outer membrane. |
| Δxy-TAILS (cm2/s) |  | Lateral diffusion coefficient in the aliphatic tail region of the outer membrane. |
| Δh-TAILS (kJ/mol) | (kJ/mol) | Interaction energy with the aliphatic tail region of the outer membrane. |
| Δs-TAILS (J/mol K) | (J/mol K) | Cumulative entropy of the drug in the aliphatic tail region of the outer membrane. |
| HB-LIPID-A |  | Total number of hydrogen bonds with the lipid-A region of the outer membrane. |
| HB-WATER-LIPID-A |  | Total number of hydrogen bonds with water molecules (if present) in the lipid-A region of the outer membrane. |
| Δxy-LIPID-A (cm2/s) |  | Lateral diffusion coefficient of the drug within the lipid-A region of the outer membrane. |
| Δh-LIPID-A (kJ/mol) | (kJ/mol) | Total interaction energy (Coulomb, Lj) with the lipid-A region of the outer membrane. |
| Δs-LIPID-A (J/mol K) | (J/mol K) | Cumulative entropy of drug in the lipid-A region of the outer membrane. |
| HB-CORE-1 |  | Total number of hydrogen bonds with the core-1 region of the outer membrane. |
| HB-WATER-CORE-1 |  | Total number of hydrogen bonds with water molecules (if present) in the core-1 region of the outer membrane. |
| Δxy-CORE-1 (cm2/s) |  | Lateral diffusion coefficient of drug within the core-1 region of the outer membrane. |
| Δh-CORE-1 (kJ/mol) | (kJ/mol) | Total interaction energy (Coulomb, LJ) with the core-1 region of the outer membrane. |
| Δs-CORE-1 (J/mol K) | (J/mol K) | Cumulative entropy of drug in the core-1 region of the outer leaflet. |
| HB-CORE-2 |  | Total number of hydrogen bonds with the core-2 region of the outer membrane. |
| HB-WATER-CORE-2 |  | Number of hydrogen bonds with water molecules (if present) in the core-2 region of the outer membrane. |
| Δxy-CORE-2 (cm2/s) |  | Lateral diffusion coefficient in the core-2 region of the outer membrane. |
| Δh-CORE-2 (kJ/mol) | (kJ/mol) | Total interaction energy with the core-2 region of the outer membrane. |
| Δs-CORE-2 (J/mol K) | (J/mol K) | Cumulative entropy of drug in the core-2 region of the outer membrane. |
